# Integrating NMR Restraints into Coarse-Grained Simulations: Toward Accurate Conformational Ensembles of Complex Protein Systems

**DOI:** 10.1021/jacs.5c22987

**Published:** 2026-03-19

**Authors:** Mina Cullen, Carmen Biancaniello, Katerina Taškova, Vedran Miletić, Davide Mercadante, Alfonso De Simone

**Affiliations:** † School of Chemical Sciences, 1415 the University of Auckland, Auckland 1142, New Zealand; ‡ Department of Pharmacy, 9307 University of Naples Federico II, Naples 80131, Italy; § Department of Computer Science, The University of Auckland, Auckland 1142, New Zealand; ∥ Max Planck Computing and Data Facility (MPCDF), Garching, Munich 85748, Germany; ⊥ Maurice Wilkins Centre for Molecular Biodiscovery, the University of Auckland, Auckland 1142, New Zealand

## Abstract

Structural dynamics play critical roles for the biological activity of protein molecules. Characterizing the inherent conformational landscapes of these macromolecules remains a major experimental and computational challenge, particularly for heterogeneous and transient systems such as intrinsically disordered proteins, membrane-associated assemblies and disordered fuzzy coats of amyloid aggregates. In this context, coarse-grained (CG) molecular dynamics simulations have enabled accessing to extended time scales and large system sizes, however, their reduced resolution and simplified interaction potentials often limit the structural accuracy. Here, we introduce Martini3-NMR, an integrative framework that incorporates nuclear magnetic resonance (NMR) observables directly into CG protein force fields. Using artificial neural networks to model NMR chemical shifts at the CG level, and integrating these data with NOE restraints, we define an approach to significantly enhance the accuracy of CG simulations while maintaining their elevated sampling efficiency, thereby resulting in a substantially improved description of protein conformational ensembles. We demonstrate the broad applicability of Martini3-NMR by generating CG ensembles for a range of systems involved in diverse biological processes such as protein folding, oligomer disassembly within lipid bilayers and conformational transitions of disordered fuzzy regions decorating amyloid fibril surfaces, which were found to display condensate-like properties. By enabling an experimentally driven and computationally efficient exploration of protein conformational landscapes, Martini3-NMR provides a novel general framework for investigating dynamic, heterogeneous and multiscale biomolecular processes. This approach opens to significant new opportunities for extending CG simulations toward a more quantitative understanding of the relationship between molecular structure, dynamics and biological function.

## Graphical abstract

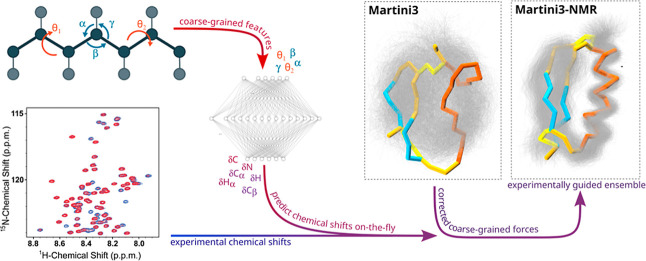

## Introduction

Structural fluctuations are fundamental determinants of the biological activity of proteins, as these biomolecules populate broad ensembles of interconverting conformations, encompassing both folded and intrinsically disordered native states.^1^ These structural dynamics span a wide range of time scales, from picoseconds to seconds, and enable proteins to access the specific conformations required for a wide spectrum of biological processes, including enzymatic catalysis,^2^ oligomeric assembly,^3^ molecular recognition and signaling,^4^ and others. A detailed characterization of these motions is critical for elucidating how proteins engage their interaction partners and dynamically access distinct functional states. Characterizing conformationally heterogeneous proteins remains a major experimental challenge, particularly when the relevant states are sparsely populated or transient in nature. This major limitation hampers the study of biochemical mechanisms that are governed by low-population protein states accessed only transiently through their inherent conformational energy landscapes. Such intermediates often play a decisive role in processes such as protein folding, conformational switching, allosteric regulation, enzymatic turnover and the assembly or disassembly of biomolecular complexes, however, their short lifetimes, intrinsic dynamics and minimal abundance render them exceptionally challenging to characterize at high resolution using conventional structural biology approaches.

When studying intermediate states that exist in dynamic equilibrium with ground-state conformations, nuclear magnetic resonance (NMR) spectroscopy has emerged as a uniquely powerful experimental approach, enabling access to a broad range of time scales and spatial resolutions.^5,6^ This technique can provide detailed structural, thermodynamic, and kinetic information through the detection of time- and ensemble-averaged observables that are inherently sensitive to conformational fluctuations. Through its evolution into solution-state and solid-state methodologies, NMR has progressively expanded its scope to encompass a wide range of systems, including cytosolic proteins, membrane proteins,^7,8^ insoluble amyloid fibrils,^9–12^ megadalton complexes^13,14^ and biomolecular condensates.^15,16^ In parallel, theoretical approaches have become increasingly important for the investigation of protein structural fluctuations at high resolution, with molecular dynamics (MD) simulations assuming a central role in this area. When integrated with NMR data as experimental restraints, the intrinsic limitations of MD force fields can be substantially mitigated, resulting in improved accuracy in the determination of dynamic conformational ensembles.^5,17,18,32^ Over the past decades, several powerful refinement strategies have been proposed to incorporate NMR restraints into full-atom MD frameworks, achieving remarkable success in improving structural accuracy and capturing local and global dynamical properties.^18–22^ Nevertheless, the inherent limitations of all-atom MD in sampling large conformational phase spaces on biologically relevant time scales restrict its applicability to the study of highly heterogeneous and dynamic systems.

Here, we present a study reporting substantial advance in the conformational sampling of proteins by introducing NMR restraints into coarse-grained (CG) models within a simulation framework designated as Martini3-NMR. CG simulations offer strategic advantage for exploring extensive conformational landscapes and accessing long time scales that are typically inaccessible to all-atom approaches. By integrating the experimental accuracy of NMR restraints with the enhanced sampling efficiency afforded by CG simulations, we show that complex biochemical processes can be characterized with unprecedented level of detail, thereby extending the scope and applicability of both techniques. We provide additional support for these conclusions by showing that NMR chemical shifts (CS) and Nuclear Overhauser effect (NOE) restraints applied within the Martini3 CG model enable accurate sampling of the conformational space of both soluble and membrane proteins, while also capturing complex processes such as the functional disassembly of oligomeric proteins within the lipid bilayers and the delineation of the topological and structural properties of disordered fuzzy coats in amyloid fibrils.

## Results

### NMR Chemical Shift Calculations in Coarse-Grained Protein Structures

We developed artificial neural networks (ANNs) to predict NMR CS of proteins from CG structural models. The ultimate goal of our work is to incorporate this model, namely NapShift-CG, as an experimental NMR-derived restraining potential into CG simulations, thereby enhancing both sampling efficiency and the structural accuracy of the resulting conformational ensembles. ANNs were generated to account for the topology of the Martini3 CG force field by deriving internal parameters from the Cartesian coordinates of the backbone (BB) and side chain (SC) beads (Figure 1a). More specifically, for each residue of the protein, NapShift-CG employs three bond angles centered on a BB (α, β, γ) and two dihedral angles involving the flanking BBs (θ_1_ and θ_2_). This topological definition makes NapShift-CG applicable to other Martini force fields^23–25,70^ including dry Martini and pol-Martini, upon reparametrization of the ANN. The method was designed to account for NMR CS of six distinct protein atoms from the main chain (N, C_α_, C, HN and H_α_) and side chains (C_β_). Instead of generating six individual ANNs where each protein atom is treated separately, as previously developed for the full-atom NapShift predictor,^26^ in NapShift-CG all six atom types are trained simultaneously. We evaluated different structural parameters derived from mono-, tri-, penta-, and heptapeptides to contribute input vectors for ANN training (Figure 1), and found that tripeptides generate the best compromise between accuracy of NapShift-CG and computational complexity. ANNs training was performed using 2986 protein structures for which CS assignments were available. A set of 250 entries from the initial database was not included in the training data set and was used as a validation data set (see the Supporting Information spreadsheet).^70^

**Figure 1:**
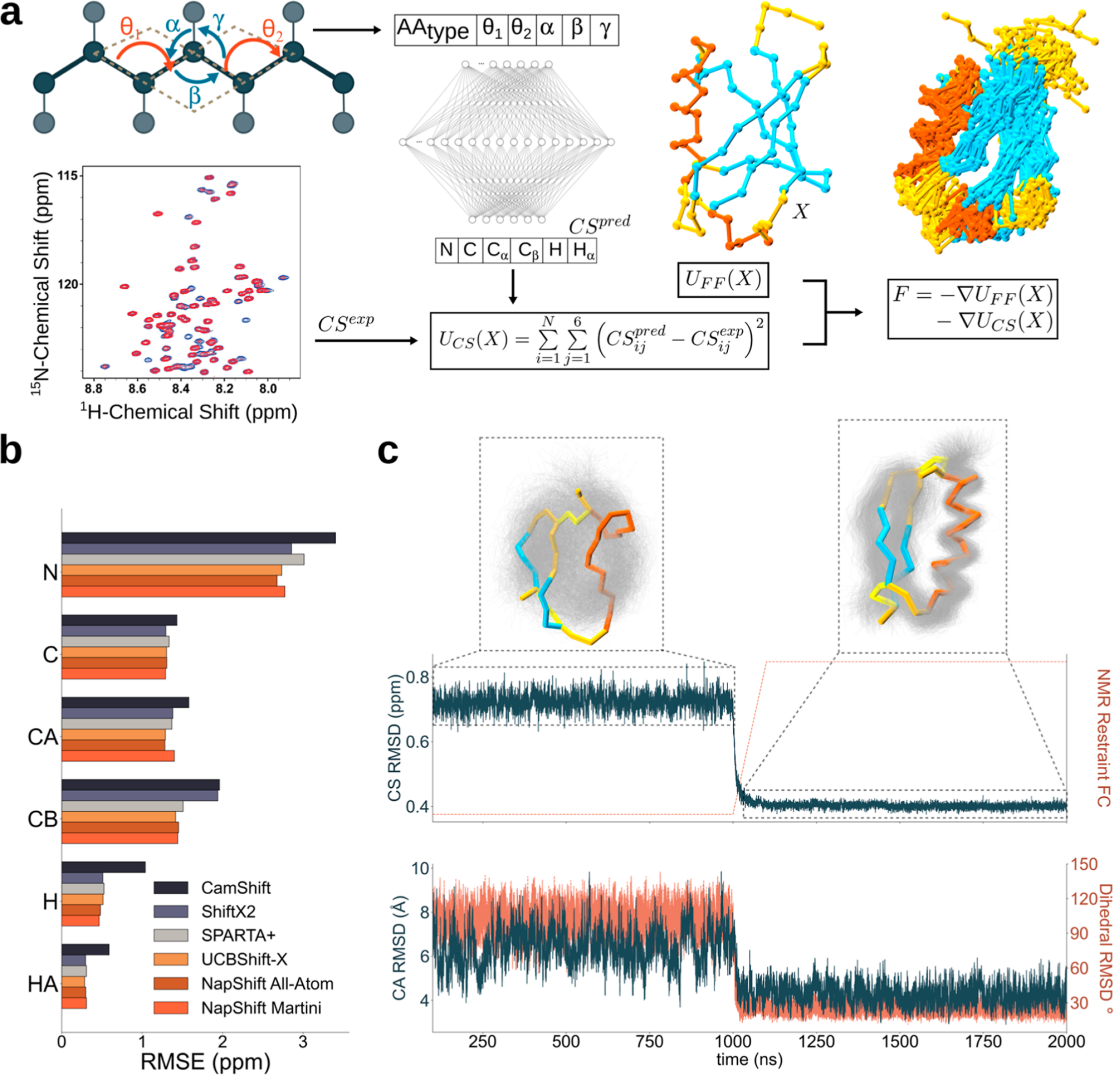
Conceptualisation, implementation and testing of Martini3-NMR. (a) Coarse-grained heptapeptide mapped with Martini3. Backbone and side chain beads are in dark and light gray, respectively. The dihedrals θ_1_ and θ_2_ and the angles α, β, and γ are the Napshift-CG inputs. Napshift-CG predicts chemical shifts (CS) for backbone protein atoms N, C, C_α_, C_β_, H and H_α_. Predicted (CS^pred^) and experimental (CS^exp^) chemical shifts are compared at each simulation step, and their difference is used to correct the discrepancy by adjusting the forces acting on the beads and to obtain CG protein ensembles, in line with experimental NMR data. On the right-hand side the CG experimental structure and the obtained ensemble of ubiquitin are colored by secondary structure (α-helices-orange, β-strands-cyan, loops-yellow). The system was simulated with *K*_CS_ = 0 for 1 μs, after which *K*_CS_ was increased to 25 over 100 ns, then simulated for a further 1 μs. The first 100 ns were discarded as equilibration time. (b) Root mean square error (RMSE) reporting on the predictive ability of each backbone atom by Napshift-CG and a series of all-atom predictors. (c) Simulation of a de novo designed mini-protein (PDB ID: 2ND3) before and after the application of NMR CS restraints. Top and bottom panel show the root-mean-square deviation (RMSD) in CS (top), dihedral and coordinates (bottom) space. The insets show the obtained ensembles (gray) before (left) and after (right) the application of the restraints. The ensemble centroids are overlaid on the ensemble and colored by secondary structure as in (a).

With this architecture, the accuracy of NapShift-CG resulted of similar magnitude to that of methods based on full-atom structures (Figure 1b). This result holds strategic relevance, as it established that CS of proteins can be predicted from CG models with state-of-the-art accuracy, thereby opening the way to several advanced applications in structural refinement and MD simulations.

### Definition of NMR-Restrained Coarse-Grained Simulations of Proteins

The architecture of NapShift-CG was based on structural parameters that are differentiable in the Cartesian space, thereby enabling the calculation of gradients and forces driving the simulations toward an improved match between computed and experimental CS (Figures 1b and S1). CS restraints were applied to the Martini3 force field using the OpenMM software^27^ (see Methods), and were initially tested on a de novo designed mini-protein for which the experimental structure (PDB code: 2ND3) and CS (BMRB code: 26046) are available.^28^ When simulating this system, unrestrained Martini3 MD simulations provided a poor representation of its conformational sampling, reaching a C_α_ mean RMSD of 6.44 ± 0.045 Å and mean dihedral RMSD of 103.16° ± 0.62° from the experimental structure (Figure 1c). The introduction of CS restraints via a gradual increase of the applied force was found to induce a rapid reduction in the CS RMSD, indicating that the implementation of the NapShift-CG derivatives was effective. Notably, the reduction in CS RMSD was found to correspond to an improvement of the conformations sampled during the MD simulations, with mean values of C_α_ and dihedral RMSD plateauing at 4.22 ± 0.02 Å and 24.18° ± 0.21°, respectively. This result is of fundamental significance, as it demonstrates that the incorporation of NMR restraints into CG force fields, such as Martini3, can dramatically enhance the ensemble quality of simplified models, particularly with respect to the dihedral angles of the protein backbone.

We next evaluated NapShift-CG on ubiquitin, a protein system featuring a more complex topology that includes a single β-sheet of five strands (in mixed parallel and antiparallel topology), an α-helix (residues 23–34) and a 3_10_-helix (residues 56–59).^29^ Using unrestrained Martini3, CG MD simulations are unable to retain the Ub structure, with mean C_α_ and dihedral RMSD of 13.47 ± 0.12 Å and 104.49° ± 0.40°, respectively (Figure S2). When applying NapShift-CG NMR restraints, the conformational space explored improved considerably with respect to the dihedral angles (with mean RMSD of 47.37° ± 0.19°), however, the simulations could not accurately capture tertiary contacts between regions that are distant in the protein sequence, leading to a mean C_α_ RMSD of 14.02 ± 0.13 Å (Figure 2a). We therefore postulated that CS restraints in CG simulations should be combined with additional NMR observables that encode the topological features of protein structure (Figure S2). To this end, we selected distance information derived from NMR NOEs as the optimal complement to CS (see Methods). Martini3 simulations restrained only by NOEs yielded ensembles of limited quality, particularly with respect to backbone dihedral sampling (Figure S2b,c). However, the simultaneous employment of both CS and NOE restraints, here after described as Martini3-NMR, generated a significant improvement of the quality of the Ub structures sampled by CG simulations, resulting in mean values of C_α_ and dihedral RMSD of 3.20° ± 0.01° Å and 44.88° ± 0.12°. We compared the Martini3-NMR ensemble with high resolution structures of Ub generated with full-atom methods and employing high resolution NMR data such as RDC^30,31^ or ^15^N-relaxation.^32^ The analysis employed the S matrix^17^ to compare pairwise distance distributions between backbone beads in the CG ensembles and Cα atoms in the corresponding full-atom ensembles, showing that Martini3-NMR achieves significantly better agreement with full-atom ensembles than either Martini3 or Martini3-DSSP (Figure S3). In addition, by calculating the root-mean-square fluctuations (RMSF) from the BB coordinates, we observed a striking correspondence between dynamical regions of the Martini3-NMR ensemble and those identified from order parameters measured in solution NMR^32^ (Figure S4a). In contrast, RMSF values calculated on Martini3 and Martini3-DSSP ensembles are abnormally high (Figure S4b), and fail to reflect the differential flexibility of loops and secondary-structure elements.

**Figure 2:**
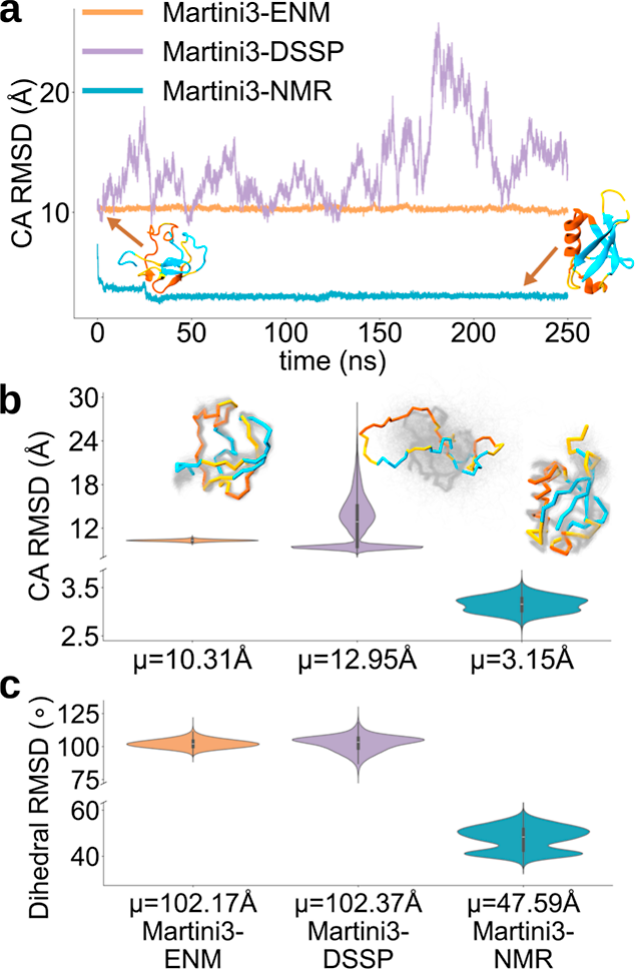
Effects of CS restraints on a partially unfolded protein. (a) Time trace of the C_α_ root-mean-square deviation obtained from simulations of ubiquitin with the initial conformation partially unfolded (C_α_ RMSD = 11.83 Å). (b,c) Coordinates (b) and dihedral (c) RMSD distributions for the simulations shown in (a). The obtained ensembles are shown above each violin plot with the distribution means (μ) reported in the tick labels.

The key aspect of Martini3-NMR lies in its ability to tailor CG-MD to specific biochemical processes, as the incorporation of system-specific NMR restraints enables the generation of ensembles that are also sensitive to specific experimental conditions under which the NMR data are acquired. Another distinct advantage of the proposed method is that it overcomes the limitations of CG approaches that restrict the conformational space of a protein to its initial coordinates, such as for example elastic network models (ENM) or Martini3-DSSP.^33^ We illustrate this fundamental point by performing CG simulations initiated from a misfolded Ub structure (Figure 2), exhibiting a C_α_ RMSD of 11.83 Å relative to the native state. When running CG simulations with elastic networks (Martini3-ENM), the conformations remained essentially frozen to the initial distorted state (C_α_ RMSD of 10.31 ± 0.01 Å), whereas the employment of Martini3-DSSP generated a variety of misfolded conformations with values of C_α_ RMSD varying from 7.99 Å to 28.79 Å along the trajectory. By contrast, when using Martini3-NMR, the system rapidly folded into the native structure, plateauing at a mean C_α_ RMSD of 3.15 ± 0.01 Å. This result highlights the fundamental improvement introduced by NMR-restrained CG simulations, which do not simply constrain the conformational space of the CG protein model but rather enhance the force field accuracy by steering the conformational ensemble toward states that are more consistent with experimental data.

Having established the optimal integration of sparse NMR data to restrain CG force fields, we tested Martini3-NMR on a variety of globular proteins of increasing structural complexity (Figure 3a). First, we tested this method on KRAS, a protein that, similarly to Ub, shows a mixed α-helical/β-sheet topology, but significantly larger than ubiquitin. KRAS is a GTPase that regulates cellular pathways involved in signaling for growth, proliferation, and differentiation. It is also the most frequently mutated oncogene in human cancer, making it an important system for structural biology. Martini3-NMR was able to retain the overall fold of KRAS with average C_α_ and dihedral RMSD values of 4.55 ± 0.03 Å and 41.53° ± 0.20°, respectively, with significant improvement compared to with Martini3 and Martini3-DSSP (Figures 3a, S5 and S6). Dynamic regions identified by elevated RMSF values in the Martini3-NMR ensemble showed a sticking agreement with highly flexible segments determined experimentally by ^15^N relaxation measurements^34^ (Figure 3b). These results highlight the ability of Martini3-NMR in capturing important dynamical properties of KRAS, including fluctuations within the switch regions. Capturing such conformational dynamics is critical, as they govern nucleotide-dependent signaling, effector recognition and regulatory interactions that determine the active and inactive states of the protein.

**Figure 3:**
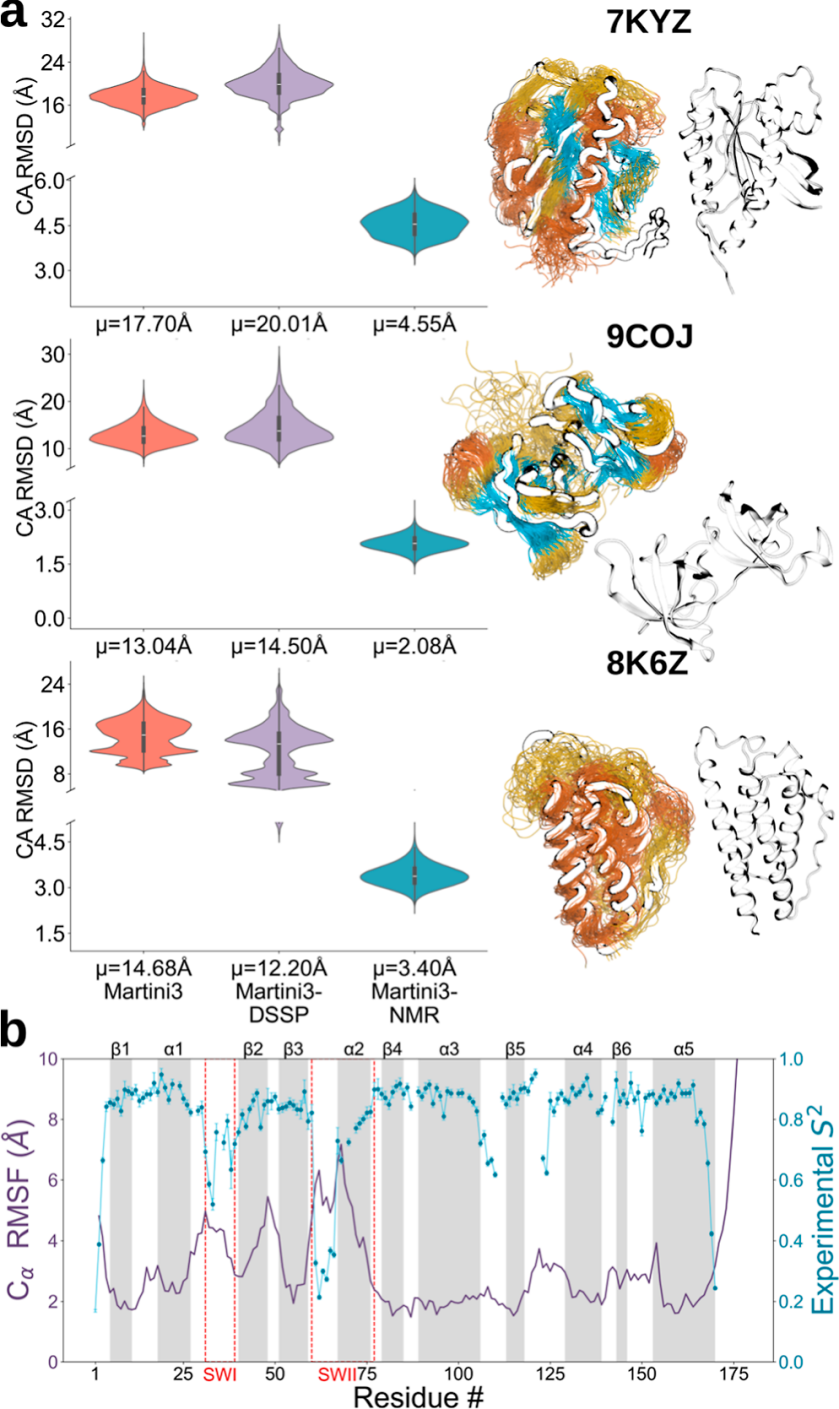
Application of Martini3-NMR to globular proteins in solution. (a,b) Representative ensembles and root-mean-square deviation (RMSD) distributions in the Cartesian space (C_α_) of soluble (a) and membrane (b) proteins. (a) Soluble proteins from top to the bottom are KRAS, the SH3 tandem domains of the human KIN protein and a human leptin. α-Helices, β-strands and loops are shown in orange, cyan and yellow, respectively. On the right side the experimental structures are reported, while on the left side the ensembles overlaid on the centroid conformations are shown. (b) Root mean square fluctuations (RMSF) calculated for the backbone beads of the Martini3-NMR KRAS ensemble, where higher RMSF values indicate regions with higher conformational flexibility. These regions show a striking correspondence with segments echibiting low order parameters (S^2^) in solution NMR ^15^N relaxation experiments of KRAS.^34^

A similar high-quality conformational ensemble was generated for the SH3 tandem of the human KIN protein (C_α_ RMSD of 2.08 ± 0.01 Å and mean dihedral RMSD of 38.95° ± 0.33°, Figures 3a, S5 and S6), a system of increased structural complexity owing to the presence of two intertwined domains. We also generated the NMR-guided CG ensemble of the human leptin, a multipotency cytokine that regulates various physiological functions and whose structure is composed of a mainly five-helix bundle structure with two hydrophobic cores. The method generated an exceptionally good ensemble of leptin with mean C_α_ RMSD of 3.40 ± 0.02 Å and mean dihedral RMSD of 32.91° ± 0.23° (Figures 3a, S5 and S6). The ensemble was also able to capture the conformational properties of intrinsically disordered regions (IDRs) in the loop connecting helices A and B as well as the flexible helix E residing in the long loop connecting helices C and D, by improving the conformational space with respect to the unrestrained simulations (Figure S8a,b). Both dynamic regions are essential for the conformational recognition of leptin by its receptor. In the Martini3-NMR ensemble, these regions are described with accuracy in terms of the backbone dihedrals, showing a remarkable improvement relative to the Martini3 ensemble thereby enabling a realistic description of the local conformational properties. This enhanced treatment of dihedral space allows the dynamic elements involved in receptor recognition to be properly sampled, suggesting that the molecular mechanisms underlying key signaling pathways can be effectively investigated using this hybrid CG-NMR force field.

### Conformational Ensembles of Membrane Proteins with Martini3-NMR

In addition to soluble proteins, we leveraged the capability of Martini3-NMR to model biological membranes and applied this approach on some relevant membrane-protein systems with increasing topological complexity (Figure 4a). First, we simulated the homodimeric transmembrane (TM) domain of the EGFR HER1, whose structure was originally resolved by solution NMR in micelles.^35^ The conformational transition between inactive and active dimer configurations of the HER1 TM domain controls receptor signaling in functional and pathological conditions such as cancer.^36^ We first modeled the structure of the TM domain by equilibrating it in a physiological lipid bilayer composed of 400 DLPC lipids (20 × 20 nm along the X and Y dimensions) as described in Thomasen et al.^37^ and to match experimental conditions.^38^ The simulations with Martini3-NMR sampled the dynamics of the inactive conformation of the TM dimeric domain by yielding a mean C_α_-RMSD of 4.21 ± 0.04 Å and mean dihedral RMSD of 43.07° ± 0.3° (Figure S6). The analysis of the interhelical tilt angle revealed that the relative orientation of the two helices yields a dimer stably sampling the inactive state, with the most populated conformation matching the experimental value of the tilt angle. Nonetheless, the distribution of tilt angles shows that Martini3-NMR also samples a minor population at negative angle values, suggestive of a pathway leading to an active state (Figure S9).

**Figure 4:**
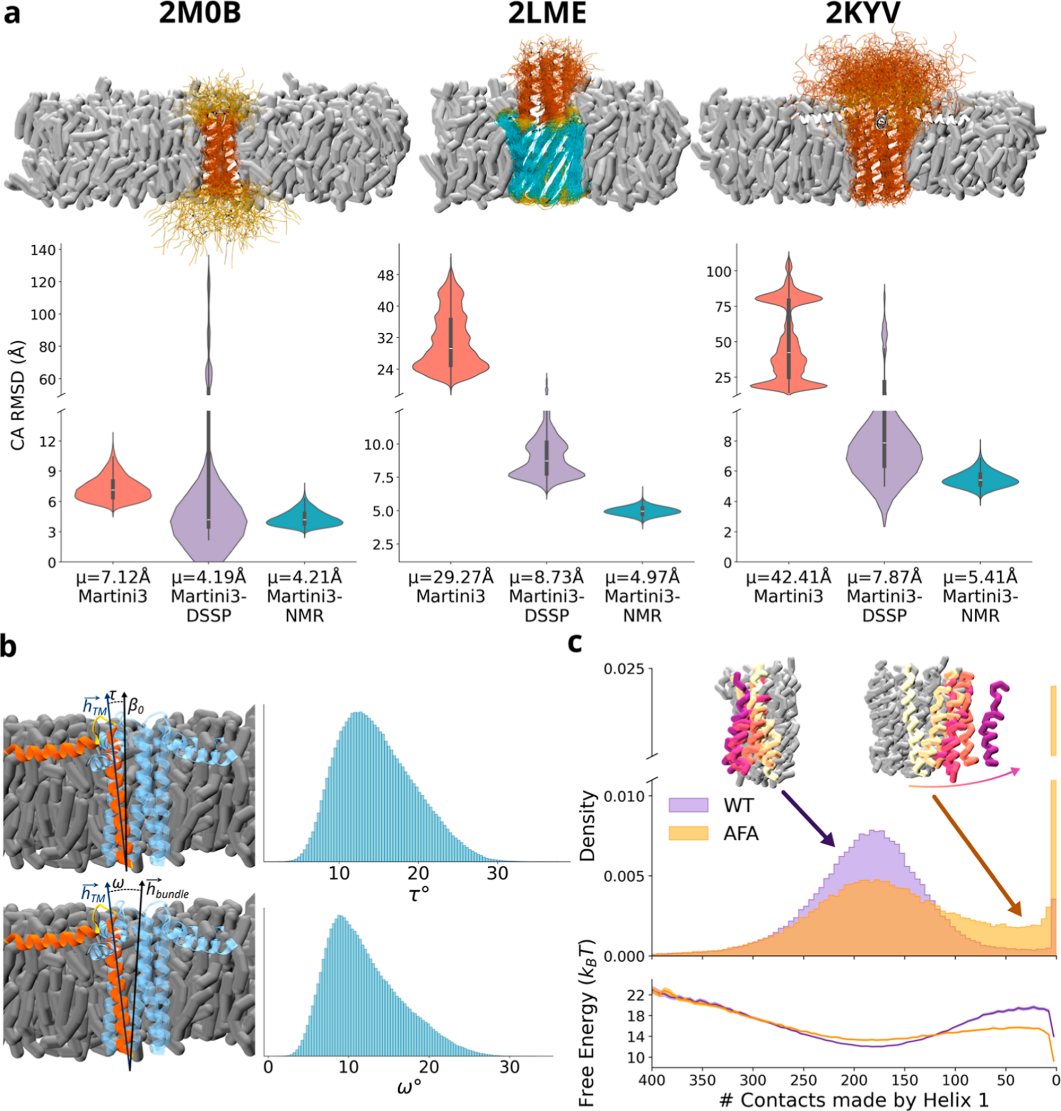
Martini3-NMR recapitulates the conformational features of membrane proteins. (a) Membrane proteins from left to right are the single-span transmembrane helical domains of the human tyrosine kinase ErbB1, the transmembrane anchor domain of the bacterial autotransporter YadA, and the phospholamban pentamer. PDB codes are reported on top of the ensembles. α-Helices, β-strands and loops are shown in orange, cyan and yellow, respectively. (b) Phospholamban pentamer in a lipid membrane (gray). One of the helices, designated as helix 1, is shown in orange while the others are shown in blue, to highlight the angles τ and ω (top and bottom panels, respectively) used to describe the conformational dynamics of the pentamer as in Sanz-Hernandez et al.^44^ The distributions of τ and ω angles yielded by Martini3-NMR simulations are shown. These angles describe the orientation of phospholamban helices with respect to either the membrane norm (τ) or the helix bundle (ω). (c) To compute the distributions of contacts of helix 1 with the remainder of the pentamer, 200 simulations each of 500 ns of WT and AFA mutant phospholamban were performed, restraining the backbone bead of the last residue to an upper limit of 3.8 nm along the z-dimension. In the top panel, the number of contacts between helix 1 and the remainder of the pentamer molecules is shown in indigo and orange for PLN^WT^ and PLN^AFA^, respectively. In the bottom panel the free energy landscape along the same reaction coordinate has been computed using the Boltzman equation.

We next investigated the TM domain of the *Yersinia enterocolitica* adhesin A (YadA), whose structure has been resolved in a lipid bilayer by solid-state NMR.^39^ This membrane domain exhibits a more complex topology, consisting of a homotrimer forming a 12-stranded membrane β-barrel, with each monomer contributing four antiparallel strands that assemble into a single symmetric pore. In this structure, each monomer also features an additional short N-terminal α-helix that extends into the aqueous phase (Figures 4a, S5 and S6). CG simulations of YadA yielded a mean RMSD of 4.97 ± 0.02 Å for the β-barrel and 6.44 ± 0.03 Å when including the incomplete helical region. Although these RMSD values are higher than those obtained for other systems examined, they remain substantially lower than those produced using Martini3 and Martini3-DSSP, indicating a significant improvement introduced by the incorporation of NMR restraints (Figure 4a).

As an additional membrane protein system, we studied the pentameric assembly of the phospholamban protein (PLN) (Figure 4a–c).^40^ This single-pass TM protein plays a critical physiological role by regulating the SERCA (sarcoplasmic/endoplasmic-reticulum Ca^2+^-ATPase) pump, thereby regulating cardiac muscle relaxation and contractility. In its physiological form, PLN exists in equilibrium between pentameric and monomeric states, with the oligomer believed to act as a reservoir for the inhibitory monomeric state, which is the form that directly interacts with SERCA.^41^ We generated the structural ensemble of the pentameric PLN using Martini3-NMR starting from a hybrid solution and solid-state NMR structure resolved in lipid bilayer.^39^ The ensemble exhibited high accuracy in modeling the TM region of the oligomer, as it successfully reproduced the orientations of the homopentameric helical bundle (residues 24 to 52), in close agreement with the full-atom ensemble previously obtained from orientational restraints and conveying the packing of the bundle identified as stabilizing components of PLN multimeric state and relevant for the general design of stably packed TM helical domains (Figure 4b),^40^ ^,^ ^42,43^ In contrast, the N-terminal membrane-surface helices resulted poorly associated with the surface of the lipid bilayer and as a consequence adopted random orientations. This behavior is likely attributable to intrinsic limitations of the CG force field in modeling protein—membrane interactions of amphipathic helices placed on top of the lipid bilayer. This limitation could be mitigated through the inclusion of orientational restraints, which are currently not implemented in Martini3-NMR.^46 37^

In addition to describing the topological-dynamical properties of the pentamer barrel, the NMR-guided CG simulations enabled sampling of the process of monomer detachment from the pentamer assembly (Figure 4c). Several lines of evidence exist about the intrinsic stability of the pentameric state of PLN with respect to the isolated monomers.^40,45^ By mutating the three Cys residues of the TM region into Ala 36, Phe 41 and Ala 46 (PLN^AFA^),^46^ it is possible to invert the energy landscape and push the equilibrium toward the monomeric state. We therefore exploited the sampling ability of CG simulations to study the monomer–pentamer equilibrium in PLN^WT^ and PLN^AFA^ (see methods). The data showed a marked difference between the two sequences when sampling a reaction coordinate of monomer detachment. In particular, while the WT stably sampled the pentameric state, with minor events of detachment, PLN^AFA^ sampled numerous trajectories of monomer detachment from the four remainder molecules, experiencing 70% detachment events vs 15% of the WT. The analyses indicate that, along a reaction coordinate based on the number of contacts between the detaching monomer and the remainder of the molecules composing the pentamer, the free energy of PLN^WT^ bound state is lower than that of the detached state. The scenario is completely inverted for PLN^AFA^ where the free energy of a configuration featuring a detached monomer from the remainder four monomers is lower than that of the assembled pentamer (Figure 4c). In addition, the AFA triple mutation also reduces the energy barrier of detachment to 2.44 ± 0.19 kJ/mol from 7.63 ± 0.19 kJ/mol of the WT. Notably, using the same chemical-shift and NOE restraint setup into full-atom simulations does not result in pentamer dissociation for either the WT or mutant systems (Figure S10), underscoring the enhanced sampling capabilities afforded by the NMR-restrained coarse-grained model. Collectively these data show that Martini3-NMR is particularly suitable for studying relevant biochemical processes in membrane proteins, such as the disassembly of the PLN pentamer along the regulation process of SERCA.

### Elucidating the Properties of Amyloid Fibrils and their Fuzzy Coats Using NMR Restrained CG

Given its suitability to investigate large molecular systems, we next assessed the ability of Martini3-NMR to simulate large protein assemblies such as amyloids. To this end, we simulated the protease-resistant fragment (residues 297–391) from the Alzheimer’s disease Tau core, assembled into a nontwisted amyloid (Figure 5a).^47^ ssNMR studies identified a rigid amyloid core composed of residues 305–357, with residues outside this region remaining highly flexible. Because the Tau_297–391_ fibrils showed no detectable twist in TEM, precluding the use of helical reconstruction methods, solid-state NMR was crucial for resolving their molecular structure. Our Martini3-NMR CG simulations retained the structural properties of the fibrillar region of the core (residues 305–357) with mean C_α_ RMSD of 5.64 ± 0.01 Å and dihedral RMSD of 57.47° ± 0.09° (Figure S11). When simulating with Martini3 or Martini3-DSSP the fibril core rapidly disassembled yielding nonviable simulations. In addition, beyond maintaining fibril stability, Martini3-NMR simulations were able to retain the network of key intersheet interactions, including Y310-V337, H329-D348, L325-V350, and L325-I354, which collectively lock the opposing β-sheets into a compact and highly stable amyloid core. The symmetric E338-E338 interface was however not well accounted, likely because protonation states of Glu residues were not defined in the featurisation of the ANN due to the lack of a sufficient amount of specific data (Figure 5b).

**Figure 5:**
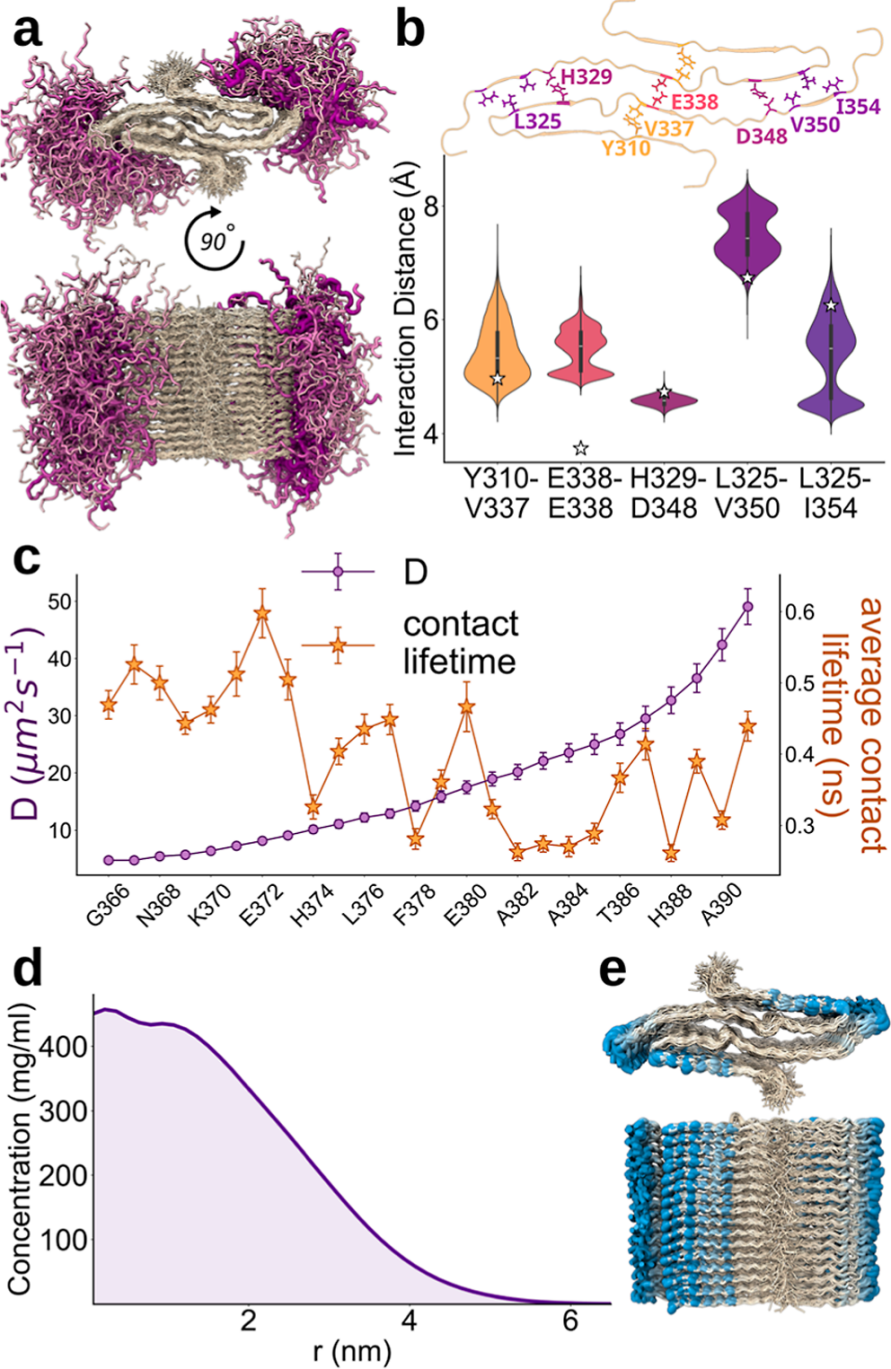
The role of the fuzzy coats in the conformational ensemble of a nontwisting tau-amyloid. (a) Ensembles of the 297–391 tau amyloid as sampled by Martini3-NMR. Core residues are colored white and the residues of the intrinsically disordered regions flanking the core (G366 to E391) are shown using a worm-like representation. The width and the color intensity of the chain are proportional to the diffusion coefficients shown in panel c. (b) Distance distributions (as violin plots) of the interacting residues deemed as stabilizing the tau amyloid formed by residues 297–391.^47^ The stars show the center of mass distance calculated from the NMR-resolved structure of the fibril (PDB ID: 8G58). (c) Residue-specific diffusion coefficients (indigo) and contact lifetimes (orange) calculated for the IDRs flanking the amyloid core. (d) Radial density profile of the IDRs as a function of the distance from the core. (e) Projection of the number of contacts between the IDRs and the core. For clarity, the intrinsically disordered regions are omitted.

In addition to investigating the properties of this amyloid core, we analyzed the characteristics of the flanking disordered regions. Increasing structural, kinetic, and cellular evidence supports the role of these regions, designated as ‘fuzzy coat’,^48–51^ as key regulatory interfaces that promote and modulate secondary nucleation as well as tune of intermolecular interactions in ways that strongly influence the toxicity, seeding efficiency, and overall biological behavior of amyloids. Because current high-resolution structural methods are primarily effective in resolving the ordered fibril core, the conformational properties of disordered fuzzy-coat regions have remained inaccessible, underscoring the need for new approaches capable of elucidating the nature of these heterogeneous and functionally critical regions. When examining the fuzzy coat of the Tau_297–391_ amyloid, the analyses revealed that the employment of CS restraints improves the conformational sampling of the disordered regions (Figure S8c). In addition, the pattern of contacts between fuzzy coats and structured regions resulted largely preserved across the disordered sequence (Figure S11b), with only partial reduction of the average contact lifetime in the C-terminal moiety of the sequence compared with the N-terminal residues lying in closer proximity to the structured core. By contrast, a significant difference between residues at the two extremities of the disordered sequence was found when analyzing the diffusion coefficient (*D*), with a 5-fold increase in *D* for the C-terminal residues (Figure 5c). Remarkably, the density profile of the fuzzy coat was found to resemble that of protein condensates (Figure 5d). Consistent with behavior observed in protein condensates,^52,53^ the IDR residues remained highly dynamic despite their elevated local concentration near the fibril core, forming numerous yet transient interactions with the core (Figure 5e) and with other disordered segments.^44^ Overall, by implementing CS and NOE restraints Martini3-NMR was able to recapitulate critical interactions and dynamics in an otherwise challenging system for coarse-grained models.

## Discussion

It is now widely recognized that structural dynamics in proteins are a key modulator of biological activity. Their role is particularly critical for intrinsically disordered proteins, whose interactions, phase-separation behavior and a wide range of other biomolecular properties depend predominantly on their inherent conformational flexibility. Despite this central relevance, protein dynamics remain challenging to characterize at a molecular level. In this context, CG simulations provide a powerful framework for sampling the conformational space of biomacromolecules, enabling access to time scales and system sizes that are prohibitive for full-atom MD. Such models have greatly expanded our ability to investigate large-scale molecular motions, supramolecular assemblies and long-time scale events that are central to biological function.^54,55^ However, the reduced representation of the system and simplified interaction potentials that underpin CG models introduce intrinsic limitations, often leading to the sampling of conformational states that are unlikely to be significantly populated under physiological conditions.

Here, we demonstrate that artificial neural networks can be employed to develop a methodology for modeling NMR chemical shifts within CG protein force fields, thereby enabling the incorporation of these data as experimental restraints to substantially improve the conformational description of proteins in such simplified representations. This integrative approach broadens the scope of molecular dynamics simulations by combining the enhanced sampling efficiency of CG models with the structural accuracy afforded by NMR restraints. We illustrate the impact of this strategy by generating CG conformational ensembles for a diverse range of systems, including soluble proteins, membrane proteins, amyloid assemblies and intrinsically disordered protein regions. The quality of the resulting coarse-grained sampling across systems spanning ∼5 kDa to ∼320 kDa demonstrates that this approach is broadly applicable to a wide range of biologically relevant systems.

The initial application of NapShift-CG to soluble systems, such as a de novo-designed mini-protein, yielded excellent agreement with experimental observations. However, for systems featuring more complex topologies, such as ubiquitin, the application of chemical shift restraints within the Martini3 framework primarily improved backbone dihedral sampling, while tertiary contacts were not fully preserved. By contrast, the simultaneous incorporation of NOE and chemical shift restraints led to a marked improvement in simulation quality, resulting in substantially enhanced representations of both protein structure and dynamics for systems such as Ubiquitin, KRAS and similar proteins. We found that Martini3-NMR is able to accurately capture the conformational dynamics of the switch regions in KRAS, thereby opening to new opportunities to investigate how oncogenic mutations, regulatory interactions and therapeutic ligands reshape signaling-relevant conformational ensembles in cancer-related processes.

The enhanced capabilities of Martini3-NMR are further illustrated by simulations initiated from a misfolded conformation of ubiquitin, which rapidly folded into the native structure and subsequently explored a conformational ensemble closely aligned with the experimental reference throughout the remainder of the simulation. In contrast, simulations performed using elastic-network Martini3 remained trapped in the misfolded state, while Martini-DSSP produced a heterogeneous set of misfolded, molten-globule–like conformations. The breadth of applications of Martini3-NMR is additional substantiated by its ability to capture the disassembly mechanism of pentameric phospholamban (PLN) within a lipid bilayer. This result demonstrates that complex processes occurring in situ in biological membranes, including the organization and rearrangement of protein assemblies, can be effectively investigated using the proposed approach. Another relevant application of Martini3-NMR concerns the characterization of the fuzzy regions of amyloid fibrils, which constitute critical sites for their propagation and biological activity. Our results indicate that these regions exhibit spatial organization, density, and dynamic properties reminiscent of protein condensates, highlighting their potential role as functionally relevant, phase-separation-like environments at the fibril surface.

In conclusion, the methodology introduced here opens new opportunities for extending CG simulations toward a more realistic and experimentally grounded description of protein conformational landscapes. By integrating NMR observables directly into CG force fields, this approach provides a general framework for interrogating dynamic, heterogeneous and multiscale processes that are otherwise difficult to access using existing computational or experimental methodologies in isolation. Martini3-NMR offers a powerful strategy for studying systems in which structural dynamics are central to function, including intrinsically disordered proteins, membrane-associated assemblies, amyloids and phase-separated condensates. Further extensions of this framework include the application of ensemble averaging to account for large scale conformational exchange across multiple conformations, as well as the incorporation of additional experimental observables to address increasingly complex cellular environments. These developments are expected to enable CG simulations to assume a more quantitative role in linking molecular structure and dynamics to biological function.

## Materials and Methods

### NapShift ANN

We trained a feed-forward Artificial Neural Network (ANN) to predict secondary CS of the 6 backbone atoms (N, C_α_, C_b,_ C, HN and H_α_). A set of 3236 NMR structures of proteins was gathered from the Protein Data Bank (PDB), with associated CS sourced from the BioMagnetic Resonance Data Bank (BMRB).^56^ Where a given PDB file contained multiple structures, only the first (lowest energy) structure was considered. We filtered out erroneous CS entries by discarding those more than 3 standard deviations from their average value in the BMRB. Raw CS were converted to secondary CS by subtracting their random-coil CS, as calculated by CamCoil.^57^

The structural properties of each residue were analyzed using a set of angles describing its local geometry and a vector encoding its amino acid type. While the all-atom ANN CS Predictor^26^ relied on the ϕ, ψ, χ1, and χ2 angles as geometric features, these atomistic resolution observables are unavailable in MARTINI3. We therefore leveraged the angle and dihedrals between a given residue’s backbone atom (BB) and those of the residues preceding and succeeding it in the protein sequence (see angles β, θ_1_ and θ_2_ in Figure 1a). We also described the orientation of the residue’s side chain by including the angles involving the first side chain atom (SC1), termed α and γ (Figure 1a). When composing the input vector, each of these angles is represented by their sine and cosine values. Missing angles (e.g., a nonexistent θ_1_ for a C-terminal residue) were given values of 0. An embedding of amino acid type was obtained from the BLOSUM62 matrix. This contained entries for the standard 20 amino acids, plus two additional to represent oxidized cysteine (CYO) and cis-proline (PRC).

In this configuration, each MARTINI3 residue is therefore represented by a vector of 32 values (22 encoding amino-acid type, and 5 × 2 describing its angular geometry). In particular, a residue’s input vector was concatenated with those of the residues before and after in sequence to produce a tripeptide representation (vector of 32 × 3 = 96 values). The architecture of the ANN involved an input layer (size 96), a hidden layer (size 26) equipped with the Exponential Linear Unit (ELU) activation function,^58^ and an output layer (size 6) with a linear activation function, as typical of other regression models. Secondary CS for the 6 backbone atoms were predicted simultaneously.

Collectively, 272,984 tripeptides extracted from 2687 structures comprised the training set for the NapShift ANN. A Mean Squared Error (MSE) loss function and the ADAM optimizer^59^ with a learning rate of 0.001 were used to train the ANN. After each epoch, an early stopping criterion was assessed on a validation set of 32,076 tripeptides from 299 structures. This served to mitigate overfitting by halting training if the validation loss did not improve after 5 epochs. The best model obtained (according to validation performance) was tested on a set of 28,107 tripeptides extracted from the 250 test structures. Structures were not mixed across the training, validation, and test sets to avoid data leakage.

A flat-bottom harmonic restraint potential is formed by the difference between CS predicted by the ANN for a simulated conformation and those measured by NMR experiments, with the width of the flat-bottom regime given by the error of the ANN test-set predictions for a given atom-type (Figure S14)­

$$V_{CS}=K_{CS}\sum_{n=1}^{N}\sum_{a=1}^{6}{\left(\delta {CS}_{n_{a}}\right)}^{2}$$

$$\delta {CS}_{n_{a}}=\{\begin{array}{l} \left(\delta {CS}_{n_{a}}^{predicted}-\delta {CS}_{n_{a}}^{\exp erimental}\right)+\varepsilon_{a}\;\text{if}\;\delta {CS}_{n_{a}}^{predicted}-\delta {CS}_{n_{a}}^{\exp erimental}<{-\varepsilon }_{a}\\ 0\;\text{if}-\varepsilon_{a}<\delta {CS}_{n_{a}}^{predicted}-\delta {CS}_{n_{a}}^{\exp erimental}<\varepsilon_{a}\\ \left(\delta {CS}_{n_{a}}^{predicted}-\delta {CS}_{n_{a}}^{\exp erimental}\right)-\varepsilon_{a}\;\text{if}\;\delta {CS}_{n_{a}}^{predicted}-\delta {CS}_{n_{a}}^{\exp erimental}>\varepsilon_{a} \end{array}$$

where δCS^predicted^ is the secondary CS predicted by the ANN, δCS^observed^ is the experimentally observed value, ε_*a*_ is the test-set RMSE of the ANN on atom type *a*, and *K*_CS_ is the force constant of the restraint potential. For each system, the value of *K*_CS_ is chosen by performing a series of simulations 5 ns-long, at increasing values of *K*_CS_ and monitoring the effect on CS RMSE. We selected the minimum value for *K*_CS_, which produces the lowest CS RMSE (Figure S12). In general, *K*_CS_ = 25 kJ mol^–1^ was found to be an appropriate parameter selection. This procedure is key to avoid biases due to over-restraining. The CS restraint potential is slowly ‘ramped up’ by increasing *K*_CS_ from 0 by 0.001 per step until its chosen maximum value is reached. The CS restraint potential has been implemented as a plugin for the OpenMM simulation engine with GPU support.^27^

### NOE Restraints

In Martini3-NMR, tertiary contacts were accounted through the structural information provided by NOESY experiments. As in the case of the CS restraints, we first translated the interatomic distances associated with NOE data into MARTINI3-compatible distances. In particular, for a given atom pair a_i_, a_j_ with an NOE-derived interatomic distance d_ij_, and coarse-grained into MARTINI beads A_i_, A_j_ respectively, we calculated their CG NOE distance D­(A_i_,A_j_) as

$$D\left(A_{i},A_{j},a_{i},a_{j},d_{ij}\right)=\left|\vec{A_{i}a_{i}}+d_{ij}\frac{\vec{a_{i}a_{j}}}{\left|\vec{a_{i}a_{j}}\right|}+\vec{a_{j}A_{j}}\right|$$

It is common for multiple groups of atoms to contribute to the same NOE observable—for example, in protons of methyl groups, giving rise to a single NOE data. When handling this situation in atomistic-resolution simulations, as in Gromacs 2025,^60^ a single distance restraint is applied across all atoms involved, which calculates its ‘apparent distance’ as

$$\mathrm{d̂}={\left(\sum_{n=1}^{N}d{\left(a_{i_{n}},a_{j_{n}}\right)}^{-6}\right)}^{-1/6}$$

since the NOE signal itself is inversely proportional to the sixth power of the interatomic distance. We here employed a similar concept to condense multiple interatomic distances into a single CG distance

$$\mathrm{D̂}\left(A_{i},A_{j}\right)=\sum_{n=1}^{N}D\left(A_{i},A_{j},a_{i},a_{j},d_{ij}\right)\cdot \frac{{\left|\vec{a_{n_{i}}a_{n_{j}}}\right|}^{-6}}{\sum_{m=1}^{N}{\left|\vec{a_{n_{i}}a_{n_{j}}}\right|}^{-6}}$$

Even though signals from multipair atomistic NOEs are condensed into a single coarse-grain distance, there may still be multiple different experimental signals which map onto the same MARTINI3 bead pair, A_i_ A_j_—for example, one might measure H_αi_-H_αj_ and C_αi_-H_αj_, corresponding to two separate interactions between BB_i_ and BB_j_. In this case, the resultant coarse-grain distance is calculated as the average coarse-grain distance for that MARTINI bead pair

$$D^{\prime }\left(A_{i},A_{j}\right)=\frac{1}{M}\sum_{m=1}^{M}\mathrm{D̂}\left(A_{m_{i}},A_{m_{j}}\right)$$

Finally, we considered the case where more than one residue pair contributes to an NOE signal. In such cases, we filtered these by checking conflicting residue pairs and discarding those with an interatomic distance significantly larger than that reported by the NOE.

Having obtained CG NOE distances, we apply the potential proposed by Torda and van Gusteren^61^

$$V_{NOE}=\{\begin{array}{l} \frac{1}{2}K_{NOE}{\left(r-r_{0}\right)}^{2},\text{if}\;r<r_{0}\\ 0,\text{if}{\;r}_{0}\leq r<r_{1}\\ \frac{1}{2}K_{NOE}{\left(r-r_{1}\right)}^{2},\text{if}{\;r}_{1}\leq r<r_{2}\\ \frac{1}{2}K_{NOE}\left(r_{2}-r_{1}\right)\left(2r-r_{2}-r_{1}\right)\;\text{if}\;r_{2}\leq r \end{array}$$

where *r* is the distance between two MARTINI3 beads and *K*_NOE_ is the force constant of the NOE restraint potential. This potential includes a harmonic regime, a flat-bottom between *r*_0_ and *r*_1_ to account for uncertainty in the measurement, and a linear regime beyond *r*2. Atomistic *r*_0_, *r*_1_ and *r*_2_ can usually be read directly from NOE data files, but where *r*_0_ and *r*_2_ are missing, we set *r*_0_ = 0 (to avoid imposing undue bias on the system) and *r*_2_ = *r*_1_+0.5 nm.

We also considered the possibility that NOESY data may imply distance restraints which are unable to be satisfied instantaneously, corresponding to different structural states experienced by the system. To account for this, we applied NOE restraints to time-averaged distances instead of instantaneous distances, as in Torda et al.,^62^ resulting in a restraining force of

$$F_{NOE}=\{\begin{array}{l} K_{NOE}^{t}\sqrt{\left(r-r_{0}\right)\left(\mathrm{r̅}-r_{0}\right)}\frac{\delta x}{r},\text{if}\;r<r_{0\;}\text{and}\overset{®}{\;r}<r_{0}\\ -K_{NOE}^{t}\cdot \min\left(\sqrt{\left(r-r_{1}\right)\left(\overset{\overset{®}{}}{r}-r_{1}\right),}r_{2}-r_{1}\right)\frac{\delta x}{r}\;\text{if}\;r>r_{1}\text{and}\overset{®}{\;r}>r_{1}\\ 0,otherwise \end{array}$$

$$\mathrm{r̅}=\mathrm{r̅}\left(t-\Delta t\right)e^{-\Delta t/\tau }+r{\left(t\right)}^{-3}\left(1-e^{-\Delta t/\tau }\right)$$

$$K_{NOE}^{t}=K_{NOE}\left(1-e^{-t/\tau }\right)$$

where δ*x* is the direction vector between the two C_α_ beads, *t* is the current simulation step, and τ = 0.05 ps is the decay time for the exponential running average. As with the CS restraint potential, this NOE restraint potential has been implemented as a plugin for OpenMM. The complete set of NMR restraint potentials (CS and NOE) are added to the potential energy arising from the MARTINI force field to produce a total potential energy of

$$V_{total}=V_{MARTINI}+V_{CS}+V_{NOE}$$

### Data Preparation

The martinize2 script^33^ version 0.13.0 was used to map experimental protein structures to their MARTINI3 representation. The cys—auto flag was used to detect disulfide bonds within systems. Membrane systems were generated from atomistic protein structures using CHARMM-GUI’s martini-maker tool.^63^ For each protein simulated, two MARTINI3 topologies were generated: one without MARTINI3 secondary structure or scFix^64^ restraints for simulation with NMR restraints, and one with MARTINI3 restraints for secondary structure detected by DSSP^65^ and scFix applied for simulation without NMR restraints. Having mapped structures according to the MARTINI3 mapping scheme, GROMACS 2024.5 ^66^ was used to place the CG models at the center of a cubic box such that all protein atoms were at least 1.2 nm from the box boundaries. The system was then solvated with MARTINI3 water beads, after which NA^+^ CL^–^ ions were added with genion, bringing the system to the desired salt concentration, as reported in the excel spreadsheet in the Supporting Information

Parameters for NOE restraints were computed using experimental data reported and structures sourced from the PDB. CS were acquired from the corresponding BMRB entries. Where specified, the full-atom NapShift^26^ was used to generate synthetic CS from the full atom experimental structure in cases where the experimental CS data set was not available or largely incomplete.

To assess the ability of Martini3-NMR to recover native-like conformations, we generated an atomistic partially unfolded conformation for all-atom Ubiquitin as shown in Figure 2, via simulated annealing using the CHARMM36 force field.^67^ After energy minimization, the system was equilibrated in the NVT and NPT ensembles, before a simulated annealing run of 5 “cycles” was performed: for each cycle, the simulation temperature was kept constant at 300 K for 5 ns, gradually raised to 800 K over 2 ns, kept constant at 800 K for a further 5 ns, before being lowered back to 300 K over 2 ns. The final frame of this simulation was used as the “melted” initial atomistic structure.

The structure of the AFA Phospholamban mutant was obtained by mutating Cys36Ala, Cys41Phe, and Cys46Ala in each transmembrane helix of the atomistic structure in PDB ID 2KYV with ChimeraX.^68^ The same set of experimental NOEs (provided in PDB 2KYV) were used for WT and AFA Phospholamban, although their mapping and the prediction of synthetic CS were performed independently for the two initial structures. For simulations investigating the dissociation of transmembrane helix 1, all intermolecular NOEs involving transmembrane helix 1 were removed.

For the full-atom simulations of Phospholamban, the initial structures were the same as those used to set up the NMR-restrained Martini3 simulations of the WT and AFA mutant. The CHARMM-GUI bilayer-builder tool was used to embed the protein in a 4:1 POPC: POPE lipid bilayer and solvate with TIP3P-charmm water and Na^+^ and Cl^–^ ions at a salt-concentration of 150 mM. Additional ions were added according to the net charge of the system, to keep the neutrality. Identical CS restraints to those used in the NMR-restrained Martini3 PLN simulations were applied, and the same data was used to apply NOE restraints between all TM-helices except H1, whose intrachain NOEs were excluded to assess monomer dissociation. Each system was initially equilibrated at constant volume and temperature (NVT ensemble), in two stages each 125 ps-long. Temperature was kept at 298 K using a Langevin thermostat with a friction coefficient of 1 ps^–1^. In the first stage, the system was positionally restrained using force constants of 4000, 2000, and 1000 kJ mol^–1^ nm^–2^ for backbone, side chain, and lipid atom, respectively. In the second stage, those positional restraints were reduced to 2000, 1000, and 400 kJ mol^–1^ nm^–2^. Similarly, an NPT equilibration step was then performed in four stages, with pressure coupled at 1 bar every 0.2 ps and temperature kept constant at 298 K as previously described. The four stages featured simulations 125, 500, 500, and 500 ps-long with restraints reduced from 1000, 500, and 400 kJ mol^–1^ nm^–2^ to 500, 200, and 200 kJ mol^–1^ nm^–2^ in the second stage, and to 200, 50, and 40 kJ mol^–1^ nm^–2^ in the third stage, for protein backbone, side chain and lipid atoms, respectively. The fourth stage only featured positional restraints applied to the protein backbone atoms using a force constant of 40 kJ mol^–1^ nm^–2^. Production simulations (without positional restraints) were run in NPT at a temperature of 298 K and a pressure of 1 bar for 500 ns with a time step of 0.002 ps. Restrained simulations were performed with *K*_CS_ = 100 kJ mol^–1^ and a *K*_NOE_ = 25 kJ mol^–1^.

The structure of the Tau amyloid was taken from the 5-layer atomistic structure resolved by ssNMR (PDB ID 8G58). This structure was duplicated and translated 4 times to create a 20-layer fiber. NOE and CS values were also duplicated to create corresponding restraints for the generated layers. CS for this system were complemented with those generated synthetically with NapShift^26^ and CamCoil^57^ for the disordered regions. Initial structures for the disordered tails were modeled as extended conformations using the modeler tool provided by ChimeraX.^68^

### Simulations

Coarse-grained Langevin dynamics simulations were carried out by OpenMM 8.2.0,^29^ using the Langevin integrator with a time step of 10 fs and friction coefficient of 0.01 ps^–1^, under periodic boundary conditions. All systems were simulated under the MARTINI3 force field.^69^ Gromacs topology files defining MARTINI3 systems were converted into OpenMM systems by the martini-openmm tool.^70^ At the beginning of each simulation, the system was energy-minimized by the steepest descent algorithm, followed by 500 ps of each NVT and NPT equilibration, during which protein atoms were positionally restrained. The CS restraints were then gradually imposed by increasing K_CS_ by 0.001 each step until the predetermined maximum value (reported for each system in Supporting Information spreadsheet) was reached. All systems were simulated in replicates of 3. We tested how an increasing amount of CS restraints randomly removed from the experimental set impact the quality of the resulting ensembles (Figure S13). Total equilibration and simulation times are reported in excel spreadsheet provided in the Supporting Information Convergence of simulation trajectories was assessed by applying the Augmented Dickey-Fuller (ADF) test to all timeseries data presented. ADF test statistics and p-values are reported in Supporting Information spreadsheet.

### Analysis

Backbone Cα RMSD and RMSF values (Figures 1c, 2a,b, 3a,4a) were calculated by first aligning all structures of an ensemble to the starting structure, then computing the RMSD using the BB beads in each simulation frame. Only structured regions were considered in the alignment and calculation of backbone RMSD. For the two-domain system 9COJ shown in Figure 3a, the alignment procedure was repeated considering each of the two domains separately. For the system 2KYV, backbone RMSD was calculated considering only the helical transmembrane domain.

Dihedral RMSD values (Figure 2c) were similarly computed by taking the RMSD of each backbone dihedral angle with respect to the starting structure of the simulations.

CS agreement (Figure 1c) was computed per-residue by comparing experimental and calculated CS for simulations with and without NMR restraints (Figure S1) and compared via their Pearson correlation coefficients. For a given conformation, the agreement between simulated and experimental CS was condensed to a single number by computing the RMSD of simulated from experimental CS for each atom type and scaling this according to the standard deviation for CS of this atom type observed in the BMRB.

Pseudo-Ramachandran plots (Figures S2c,S8) were obtained by calculating and plotting the main chain dihedral angles θ_1_ and θ_2_ (Figure 1a) for each residue of the protein and across the ensemble structures.

τ and ω angles (Figure 4 were computed as in Sanz-Hernandez et al.^44^ using the vectors *B*_0_ (the membrane normal), ${\mathrm{h⃗}}_{TM_{i}}$ (the direction vector of a single transmembrane helix (i), and ${\mathrm{h⃗}}_{bundle}$ (the direction vector of the entire transmembrane helical bundle). ${\mathrm{h⃗}}_{TM_{i}}$ was calculated by taking the difference between the positions of the backbone beads at the beginning and end of transmembrane helix *i* ( $start_{TM_{i}}$ and $end_{TM_{i}}$ ), and ${\mathrm{h⃗}}_{bundle}$ was calculated as

$${\mathrm{h⃗}}_{bundle}=\frac{1}{5}\sum_{i=1}^{5}start_{TM_{i}}-\frac{1}{5}\sum_{i=1}^{5}end_{TM_{i}}$$

and subsequently compute

$$\tau =\frac{1}{5}\sum_{i=1}^{5}angle\left(B_{0},{\mathrm{h⃗}}_{TM_{i}}\right);\omega =\frac{1}{5}\sum_{i=1}^{5}angle\left({\mathrm{h⃗}}_{bundle},{\mathrm{h⃗}}_{TM_{i}}\right)$$

The dissociation of transmembrane helix 1 from the phospholamban pentamer was calculated using the total number of contacts made by the helix with the rest of the transmembrane bundle using a cutoff of 0.8 nm to assign a contact. A conformation was considered “dissociated” if the number of contacts was found to be 0. Histograms of contact counts were transformed into free energy surfaces by calculating

$$F_{i}=-K_{B}T\ln\left(H_{i}\right)$$

where *F*_*i*_ is the free energy calculated for histogram bin *i*, *K*_B_ is the Boltzmann constant, *T* is the simulation temperature, and *H*_*i*_ is the density in bin *i*. Errors on *F* were calculated as

$$\sigma_{i}=\frac{K_{B}T}{\frac{1}{N}\sum_{n=1}^{N}H_{n_{i}}}\sqrt{\frac{1}{N-1}\left(\frac{1}{N}\sum_{n=1}^{N}H_{n_{i}}^{2}-{\left(\frac{1}{N}\sum_{n=1}^{N}H_{n_{i}}\right)}^{2}\right)}$$

where *H*_ni_ is the nth histogram of *N*bootstrap samples.

In the analysis of the tau-amyloid, the first and last two layers of the amyloid assembly (totalling 20 layers encompassing 40 chains in our model) were discarded to avoid end-effects in the fibril. Mean-square displacement (MSD) of the amyloid’s disordered tails was computed by first aligning all frames of an amyloid ensemble using the backbone beads of the structured fibril core. The MSD of a given backbone bead in a disordered tail was calculated using MDAnalysis^71^ with a minimum lag time of 10 ps (the reporting frequency of the simulation). We consider the segment between 100 and 500 ps to correspond to the linear regime of MSD. For each residue, its diffusion coefficient was computed by fitting a linear model to this linear regime. Pearson correlation coefficients for these fits are reported in the Supporting Information spreadsheet. Tail residues in the ensemble shown in Figure 5c are colored according to the natural log of their computed D.

Contact lifetime analysis employed the transition-based definition described in Galvanetto et al.,^52^ whereby a contact between two residues is considered formed when the minimum distance of all bead-pairs for these two residues drops below *r*_0_ and considered broken the next time that this minimum distance rises above *r*_1_. We set *r*_0_ = 0.8 nm, and *r*_1_ = 1.0 nm. The average contact lifetime of a given tail residue with the fiber core was calculated by taking the average of residue—residue contact lifetimes across all 36 chains, then for each tail residue taking its average contact lifetime with all core residues. Contacts that were not formed in any replicate simulation were not included in this average.

The radial concentration profile of the disordered tau amyloid tails was computed as

$$c\left(r_{i}\right)=\frac{C\left(r_{i}\right)-C\left(r_{i-1}\right)}{\pi \left(r_{i}^{2}-r_{i-1}^{2}\right)x\left(1-\theta \right)x\left(z_{\max}-z_{\min}\right)}$$

where *C*(*r*_*i*_) is the number of tail atoms less than *r*_*i*_ nm from the point of attachment to the fiber core in the *xy* plane, $\theta =\frac{\pi }{2}$ is the proportion of the circular area occupied by the structured core (which should be excluded from the density calculation), and *z*_min_, *z*_max_ are the z-coordinates of the bottom and top of the core, respectively. Tail atoms with z coordinates higher than *z*_max_ or lower than *z*_min_ were not included in the calculation.

The twist angle between successive layers of the fiber core was computed as the dihedral between the BB beads L325_a_, L325_b_, L325_c_, L325_d_ in chains a, b (layer *i*), c, d (layer *i*+1), similar to the procedure described in Periole et al.^72^ For each conformation, the average twist angle was calculated and used to produce the distributions shown in Supporting Information Figure S11.

### S Matrix

To quantify how accurately the CG ensembles match with high-resolution full-atom structural ensembles generated with experimental data, we used the S-matrix method. This metric quantifies the similarity in the pairwise distance distributions between two ensembles thereby comparing for both structure and dynamics of the two structural bundles.

For a protein of N residues, the S-matrix is an N x N matrix where each element S_*ij*_ measures the difference between the distance distributions for a given residue pair (*i*, *j*) in a reference and a simulated ensemble.

For each ensemble, we computed all pairwise distances between the backbone Cα atoms (in the full atomic ensembles) or BB particles (in the coarse grained ensembles) over all the conformers and converted these distances into normalized distributions over k bins. For a given residue pair (i,j) the S_i,j_-value is defined as

$$S_{ij}=\sum_{k}^{bins}\left|{P_{ij,k}}^{ens\;A}-{P_{ij,k}}^{ensB}\right|$$

where P_*ij,k*_is the normalized population in the bin k.

Since both distributions are normalized to 1, the S_*ij*_ values range from 0 to 2, where S_*ij*_ = 0 indicates that the two distributions are identical and S_ij_ = 2 corresponds to nonoverlapping distributions.

### Visualization

Simulated conformations were visualized using VMD version 1.9.4^74^ and ChimeraX version 1.8^70^. Coarse-grained ensembles (gray lines) in Figures 1c,2b and 3 are shown paired with a representative structure. These representative structures were obtained by performing a PCA on the ensemble coordinates and selecting the structure closest to the average of the PCA-transformed ensemble.

## Supplementary Material





## Data Availability

The excel spreadsheet named “Supporting Information.xlsx” provides the following data: The spreadsheet named “Dataset” provides the list of PDB files for the train, validation and test sets. The spreadsheet named “Systems” contains simulation conditions and DOIs for the systems simulated (membrane and soluble proteins). The spreadsheet named “ADF Statistics” provides tables of the Augmented Dickey-Fuller (ADF) coefficients calculated to assess stationarity of all the presented timeseries. Cells highlighted in red report stationarity, within a 5% confidence level, otherwise within a 1% confidence level. The spreadsheet named “MSD Correlation” provides the correlation coefficients obtained from the fit of the linear regime of the mean square displacement traces obtained for each residue shown in Figure 5c. Napshift plugins for OpenMM and Martini3-NMR tutorials are available at: https://github.com/mercadde/openmm-napshift and https://github.com/mercadde/openmm-noe

## Notes

Ubiquitin samplings; S matrices; RMSF plots; CS RMSD distributions; Dihedral RMSD distributions; Pseudoramachandran plots; Orientations of the transmembrane helices of the human tyrosine kinase ErbB1; Full atom phospholamban pentamer samplings; Twisting of the amyloid tau; Flat-bottom chemical shift restraint potential (PDF)
(XLSX)

## References

[1] Holehouse A. S., Kragelund B. B. (2024). The Molecular Basis for Cellular Function of Intrinsically
Disordered Protein Regions. Nat. Rev. Mol. Cell
Biol..

[2] Eisenmesser E. Z., Bosco D. A., Akke M., Kern D. (2002). Enzyme Dynamics during
Catalysis. Science.

[3] Karamanos T. K., Jackson M. P., Calabrese A. N., Goodchild S. C., Cawood E. E., Thompson G. S., Kalverda A. P., Hewitt E. W., Radford S. E. (2019). Structural Mapping of Oligomeric
Intermediates in an
Amyloid Assembly Pathway. eLife.

[4] Wong W., Gough N. R. (2009). Focus Issue: The Protein Dynamics of Cell Signaling. Sci. Signal..

[5] Neudecker P., Robustelli P., Cavalli A., Walsh P., Lundström P., Zarrine-Afsar A., Sharpe S., Vendruscolo M., Kay L. E. (2012). Structure of an Intermediate State in Protein Folding
and Aggregation. Science (1979).

[6] Sedinkin S. L., Burns D., Shukla D., Potoyan D. A., Venditti V. (2023). Solution Structure
Ensembles of the Open and Closed Forms of the ∼ 130 KDa Enzyme
I via AlphaFold Modeling, Coarse Grained Simulations, and NMR. J. Am. Chem. Soc..

[7] Sučec I., Xia B., Somberg N. H., Wang Y., Jo H., Li S., Perrone B., Gao Z., Hong M. (2025). Ion Channel Structure
and Function of the MERS Coronavirus E Protein. Sci. Adv..

[8] Weber D. K., Reddy U. V., Wang S., Larsen E. K., Gopinath T., Gustavsson M. B., Cornea R. L., Thomas D. D., De Simone A., Veglia G. (2021). Structural Basis for Allosteric Control of the SERCA-Phospholamban
Membrane Complex by Ca2+ and Phosphorylation. eLife.

[9] Lee M., Wang T., Makhlynets O. V., Wu Y., Polizzi N. F., Wu H., Gosavi P. M., Stöhr J., Korendovych I. V., Degrado W. F., Hong M. (2017). Zinc-Binding Structure of a Catalytic
Amyloid from Solid-State NMR. Proc. Natl. Acad.
Sci..

[10] Sahoo B. R., Genjo T., Bekier M., Cox S. J., Stoddard A. K., Ivanova M., Yasuhara K., Fierke C. A., Wang Y., Ramamoorthy A. (2018). Alzheimer’s Amyloid-Beta Intermediates Generated
Using Polymer-Nanodiscs. Chem. Commun..

[11] Tycko R. (2025). The Evolving
Role of Solid State Nuclear Magnetic Resonance Methods in Studies
of Amyloid Fibrils. Curr. Opin. Struct. Biol..

[12] Ahmed R., Akcan M., Khondker A., Rheinstädter M. C., Bozelli J. C., Epand R. M., Huynh V., Wylie R. G., Boulton S., Huang J., Verschoor C. P., Melacini G. (2019). Atomic Resolution Map of the Soluble Amyloid Beta Assembly
Toxic Surfaces. Chem. Sci..

[13] Mainz A., Religa T. L., Sprangers R., Linser R., Kay L. E., Reif B. (2013). NMR Spectroscopy of Soluble Protein Complexes at One Mega-Dalton
and Beyond. Angew. Chem., Int. Ed..

[14] Sprangers R., Kay L. E. (2007). Quantitative Dynamics
and Binding Studies of the 20S
Proteasome by NMR. Nature.

[15] Murray D. T., Kato M., Lin Y., Thurber K. R., Hung I., McKnight S. L., Tycko R. (2017). Structure of FUS Protein
Fibrils
and Its Relevance to Self-Assembly and Phase Separation of Low-Complexity
Domains. Cell.

[16] Wake N., Weng S. L., Zheng T., Wang S. H., Kirilenko V., Mittal J., Fawzi N. L. (2025). Expanding the Molecular
Grammar of
Polar Residues and Arginine in FUS Phase Separation. Nat. Chem. Biol..

[17] De
Simone A., Richter B., Salvatella X., Vendruscolo M. (2009). Toward an Accurate Determination of Free Energy Landscapes
in Solution States of Proteins. J. Am. Chem.
Soc..

[18] Camilloni C., Robustelli P., Simone A. De, Cavalli A., Vendruscolo M. (2012). Characterization
of the Conformational Equilibrium between the Two Major Substates
of RNase a Using NMR Chemical Shifts. J. Am.
Chem. Soc..

[19] De
Simone A., Aprile F. A., Dhulesia A., Dobson C. M., Vendruscolo M. (2015). Structure of a Low-Population Intermediate State in
the Release of an Enzyme Product. eLife.

[20] De
Simone A., Montalvao R. W., Dobson C. M., Vendruscolo M. (2013). Characterization
of the Interdomain Motions in Hen Lysozyme Using Residual Dipolar
Couplings as Replica-Averaged Structural Restraints in Molecular Dynamics
Simulations. Biochemistry.

[21] De
Simone A., Montalvao R. W., Vendruscolo M. (2011). Determination
of Conformational Equilibria in Proteins Using Residual Dipolar Couplings. J. Chem. Theory Comput..

[22] De
Simone A., Gustavsson M., Montalvao R. W., Shi L., Veglia G., Vendruscolo M. (2013). Structures of the Excited States
of Phospholamban and Shifts in Their Populations upon Phosphorylation. Biochemistry.

[23] De
Jong D. H., Singh G., Bennett W. F. D., Arnarez C., Wassenaar T. A., Schäfer L. V., Periole X., Tieleman D. P., Marrink S. J. (2013). Improved Parameters for the Martini Coarse-Grained
Protein Force Field. J. Chem. Theory Comput..

[24] Monticelli L., Kandasamy S. K., Periole X., Larson R. G., Tieleman D. P., Marrink S. J. (2008). The MARTINI
Coarse-Grained Force Field: Extension to
Proteins. J. Chem. Theory Comput..

[25] Marrink S. J., Risselada H. J., Yefimov S., Tieleman D. P., De Vries A. H. (2007). The MARTINI
Force Field: Coarse Grained Model for Biomolecular Simulations. J. Phys. Chem. B.

[26] Qi G., Vrettas M. D., Biancaniello C., Sanz-Hernandez M., Cafolla C. T., Morgan J. W. R., Wang Y., De Simone A., Wales D. J. (2022). Enhancing Biomolecular Simulations with Hybrid Potentials
Incorporating NMR Data. J. Chem. Theory Comput..

[27] Eastman P., Galvelis R., Peláez R. P., Abreu C. R. A., Farr S. E., Gallicchio E., Gorenko A., Henry M. M., Hu F., Huang J., Krämer A., Michel J., Mitchell J. A., Pande V. S., Rodrigues J. P., Rodriguez-Guerra J., Simmonett A. C., Singh S., Swails J., Turner P., Wang Y., Zhang I., Chodera J. D., De Fabritiis G., Markland T. E. (2024). OpenMM 8: Molecular Dynamics Simulation with Machine
Learning Potentials. J. Phys. Chem. B.

[28] Bhardwaj G., Mulligan V. K., Bahl C. D., Gilmore J. M., Harvey P. J., Cheneval O., Buchko G. W., Pulavarti S. V. S. R. K., Kaas Q., Eletsky A., Huang P. S., Johnsen W. A., Greisen P. J., Rocklin G. J., Song Y., Linsky T. W., Watkins A., Rettie S. A., Xu X., Carter L. P., Bonneau R., Olson J. M., Coutsias E., Correnti C. E., Szyperski T., Craik D. J., Baker D. (2016). Accurate de
Novo Design
of Hyperstable Constrained Peptides. Nature.

[29] Vijay-kumar S., Bugg C. E., Cook W. J. (1987). Structure of Ubiquitin
Refined at
1.8Åresolution. J. Mol. Biol..

[30] Lange O. F., Lakomek N. A., Farès C., Schröder G. F., Walter K. F. A., Becker S., Meiler J., Grubmüller H., Griesinger C., De Groot B. L. (2008). Recognition Dynamics
up to Microseconds
Revealed from an RDC-Derived Ubiquitin Ensemble in Solution. Science (1979).

[31] Montalvao R. W., De Simone A., Vendruscolo M. (2012). Determination of Structural Fluctuations
of Proteins from Structure-Based Calculations of Residual Dipolar
Couplings. J. Biomol. NMR.

[32] Lindorff-Larsen K., Best R. B., DePristo M. A., Dobson C. M., Vendruscolo M. (2005). Simultaneous
Determination of Protein Structure and Dynamics. Nature.

[33] Kroon P. C., Grünewald F., Barnoud J., van Tilburg M., Brasnett C., Souza P. C., Wassenaar T. A., Marrink S. J. (2025). Martinize2 and Vermouth Provide a
Unified Framework
for Molecular Topology Generation. eLife.

[34] Hansen A. L., Xiang X., Yuan C., Bruschweiler-Li L., Brüschweiler R. (2023). Excited-State Observation of Active K-Ras Reveals Differential
Structural Dynamics of Wild-Type versus Oncogenic G12D and G12C Mutants. Nat. Struct. Mol. Biol..

[35] Bocharov E. V., Lesovoy D. M., Pavlov K. V., Pustovalova Y. E., Bocharova O. V., Arseniev A. S. (2016). Alternative Packing of EGFR Transmembrane
Domain Suggests That Protein–Lipid Interactions Underlie Signal
Conduction across Membrane. Biochimica et Biophysica
Acta (BBA)—Biomembranes.

[36] Zanetti-Domingues L. C., Korovesis D., Needham S. R., Tynan C. J., Sagawa S., Roberts S. K., Kuzmanic A., Ortiz-Zapater E., Jain P., Roovers R. C., Lajevardipour A., van Bergen en Henegouwen P. M. P., Santis G., Clayton A. H. A., Clarke D. T., Gervasio F. L., Shan Y., Shaw D. E., Rolfe D. J., Parker P. J., Martin-Fernandez M. L. (2018). The Architecture
of EGFR’s Basal Complexes Reveals Autoinhibition Mechanisms
in Dimers and Oligomers. Nat. Commun..

[37] Thomasen F. E., Skaalum T., Kumar A., Srinivasan S., Vanni S., Lindorff-Larsen K. (2024). Rescaling
Protein-Protein Interactions
Improves Martini 3 for Flexible Proteins in Solution. Nat. Commun..

[38] Artemenko E. O., Egorova N. S., Arseniev A. S., Feofanov A. V. (2008). Transmembrane Domain
of EphA1 Receptor Forms Dimers in Membrane-like Environment. Biochimica et Biophysica Acta (BBA) - Biomembranes.

[39] Shahid S. A., Bardiaux B., Franks W. T., Krabben L., Habeck M., Van Rossum B. J., Linke D. (2012). Membrane-Protein Structure Determination
by Solid-State NMR Spectroscopy of Microcrystals. Nat. Methods.

[40] Verardi R., Shi L., Traaseth N. J., Walsh N., Veglia G. (2011). Structural Topology
of Phospholamban Pentamer in Lipid Bilayers by a Hybrid Solution and
Solid-State NMR Method. Proc. Natl. Acad. Sci.
U. S. A..

[41] Toyoshima C., Asahi M., Sugita Y., Khanna R., Tsuda T., MacLennan D. H. (2003). Modeling of the Inhibitory Interaction of Phospholamban
with the Ca2+ ATPase. Proc. Natl. Acad. Sci.
U. S. A..

[42] Mravic M., Thomaston J. L., Tucker M., Solomon P. E., Liu L., DeGrado W. F. (2019). Packing
of Apolar Side Chains Enables Accurate Design
of Highly Stable Membrane Proteins. Science
(1979).

[43] Liu W., Fei J. Z., Kawakami T., Smith S. O. (2007). Structural Constraints
on the Transmembrane and Juxtamembrane Regions of the Phospholamban
Pentamer in Membrane Bilayers: Gln29 and Leu52. Biochim. Biophys. Acta Gen. Subj..

[44] Sanz-Hernández M., Vostrikov V. V., Veglia G., De Simone A. (2016). Accurate Determination
of Conformational Transitions in Oligomeric Membrane Proteins. Sci. Rep..

[45] Funk F., Kronenbitter A., Hackert K., Oebbeke M., Klebe G., Barth M., Koch D., Schmitt J. P. (2023). Phospholamban Pentamerization
Increases Sensitivity and Dynamic Range of Cardiac Relaxation. Cardiovasc. Res..

[46] Weber D. K., Reddy U. V., Robia S. L., Veglia G. (2024). Pathological Mutations
in the Phospholamban Cytoplasmic Region Affect Its Topology and Dynamics
Modulating the Extent of SERCA Inhibition. Biochimica
et Biophysica Acta (BBA) - Biomembranes.

[47] Duan P., Dregni A. J., Mammeri N. El, Hong M. (2023). Structure
of the Nonhelical
Filament of the Alzheimer’s Disease Tau Core. Proc. Natl. Acad. Sci. U. S. A..

[48] Milanesi M., Brotzakis Z. F., Vendruscolo M. (2025). Transient
Interactions between the
Fuzzy Coat and the Cross-β Core of Brain-Derived Aβ42
Filaments. Sci. Adv..

[49] Ulamec S. M., Brockwell D. J., Radford S. E. (2020). Looking Beyond the Core: The Role
of Flanking Regions in the Aggregation of Amyloidogenic Peptides and
Proteins. Front. Neurosci..

[50] Faidon
Brotzakis Z., Löhr T., Truong S., Hoff S., Bonomi M., Vendruscolo M. (2023). Determination of the Structure and
Dynamics of the Fuzzy Coat of an Amyloid Fibril of IAPP Using Cryo-Electron
Microscopy. Biochemistry.

[51] Wegmann S., Medalsy I. D., Mandelkow E., Müller D. J. (2012). The Fuzzy
Coat of Pathological Human Tau Fibrils Is a Two-Layered Polyelectrolyte
Brush. Proc. Natl. Acad. Sci. U. S. A..

[52] Galvanetto N., Ivanović M. T., Chowdhury A., Sottini A., Nüesch M. F., Nettels D., Best R. B., Schuler B. (2023). Extreme Dynamics in
a Biomolecular Condensate. Nature.

[53] Ivanović M. T., Best R. B. (2025). All-Atom Simulations
of Biomolecular Condensates. Curr. Opin. Struct.
Biol..

[54] Pipatpadungsin N., Chao K., Rouse S. L. (2024). Coarse-Grained
Simulations of Adeno-Associated
Virus and Its Receptor Reveal Influences on Membrane Lipid Organization
and Curvature. J. Phys. Chem. B.

[55] Jefferies D., Shearer J., Khalid S. (2019). Role of O-Antigen in
Response to
Mechanical Stress of the E. Coli Outer Membrane: Insights from Coarse-Grained
MD Simulations. J. Phys. Chem. B.

[56] Ulrich E. L., Akutsu H., Doreleijers J. F., Harano Y., Ioannidis Y. E., Lin J., Livny M., Mading S., Maziuk D., Miller Z., Nakatani E., Schulte C. F., Tolmie D. E., Kent
Wenger R., Yao H., Markley J. L. (2007). BioMagResBank. Nucleic Acids Res..

[57] De
Simone A., Cavalli A., Hsu S. T. D., Vranken W., Vendruscolo M. (2009). Accurate Random Coil Chemical Shifts from an Analysis
of Loop Regions in Native States of Proteins. J. Am. Chem. Soc..

[58] Clevert, D. A. ; Unterthiner, T. ; Hochreiter, S. Fast and Accurate Deep Network Learning by Exponential Linear Units (ELUs). In ICLR 2016—Conference Track Proceedings; 4th International Conference on Learning Representations: 2015.

[59] Kingma, D. P. ; Ba, J. L. Adam: A Method for Stochastic Optimization. In ICLR 2015—Conference Track Proceedings; 3rd International Conference on Learning Representations: 2014.

[60] Abraham, M. ; Alekseenko, A. ; Andrews, B. ; Basov, V. ; Bauer, P. ; Bird, H. ; Briand, E. ; Brown, A. ; Doijade, M. ; Fiorin, G. ; Fleischmann, S. ; Gorelov, S. ; Gouaillardet, G. ; Gray, A. ; Irrgang, M. E. ; Jalalypour, F. ; Johansson, P. ; Kutzner, C. ; Łazarski, G. ; Lemkul, J. A. ; Lundborg, M. ; Merz, P. ; Miletić, V. ; Morozov, D. ; Müllender, L. ; Nabet, J. ; Páll, S. ; Pasquadibisceglie, A. ; Pellegrino, M. ; Piasentin, N. ; Rapetti, D. ; Sadiq, M. U. ; Santuz, H. ; Schulz, R. ; Shirts, M. ; Shugaeva, T. ; Shvetsov, A. ; Turner, P. ; Villa, A. ; Wingbermühle, S. ; Hess, B. ; Lindahl, E. . GROMACS 2025.1 Manual.

[61] Torda A. E., van Gunsteren W. F. (1991). The Refinement of NMR Structures
by Molecular Dynamics
Simulation. Comput. Phys. Commun..

[62] Torda A. E., Scheek R. M., van Gunsteren W. F. (1989). Time-Dependent
Distance Restraints
in Molecular Dynamics Simulations. Chem. Phys.
Lett..

[63] Qi Y., Ingólfsson H. I., Cheng X., Lee J., Marrink S. J., Im W. (2015). CHARMM-GUI
Martini Maker for Coarse-Grained Simulations with the
Martini Force Field. J. Chem. Theory Comput..

[64] Herzog F. A., Braun L., Schoen I., Vogel V. (2016). Improved Side Chain
Dynamics in MARTINI Simulations of Protein-Lipid Interfaces. J. Chem. Theory Comput..

[65] Kabsch W., Sander C. (1983). Dictionary of Protein Secondary Structure:
Pattern
Recognition of Hydrogen-Bonded and Geometrical Features. Biopolymers.

[66] Berendsen H. J. C., van der Spoel D., van Drunen R. (1995). GROMACS: A
Message-Passing Parallel Molecular Dynamics Implementation. Comput. Phys. Commun..

[67] Best R. B., Zhu X., Shim J., Lopes P. E. M., Mittal J., Feig M., MacKerell A. D. (2012). Optimization
of the Additive CHARMM All-Atom Protein
Force Field Targeting Improved Sampling of the Backbone φ, ψ
and Side-Chain Χ1 and Χ2 Dihedral Angles. J. Chem. Theory Comput..

[68] Meng E. C., Goddard T. D., Pettersen E. F., Couch G. S., Pearson Z. J., Morris J. H., Ferrin T. E. (2023). UCSF ChimeraX: Tools for Structure
Building and Analysis. Protein Sci..

[69] Souza P. C. T., Alessandri R., Barnoud J., Thallmair S., Faustino I., Grünewald F., Patmanidis I., Abdizadeh H., Bruininks B. M. H., Wassenaar T. A., Kroon P. C., Melcr J., Nieto V., Corradi V., Khan H. M., Domański J., Javanainen M., Martinez-Seara H., Reuter N., Best R. B., Vattulainen I., Monticelli L., Periole X., Tieleman D. P., de Vries A. H., Marrink S. J. (2021). Martini 3: A General Purpose Force Field for Coarse-Grained
Molecular Dynamics. Nat. Methods.

[70] MacCallum J. L., Hu S., Lenz S., Souza P. C. T., Corradi V., Tieleman D. P. (2023). An Implementation
of the Martini Coarse-Grained Force Field in OpenMM. Biophys. J..

[71] Michaud-Agrawal N., Denning E. J., Woolf T. B., Beckstein O. (2011). MDAnalysis:
A Toolkit for the Analysis of Molecular Dynamics Simulations. J. Comput. Chem..

[72] Humphrey W., Dalke A., Schulten K. (1996). VMD: Visual Molecular Dynamics. J. Mol. Graph..

