# Supplementary material for: Integrating NMR Restraints into Coarse-Grained Simulations: Toward Accurate Conformational Ensembles of Complex Protein Systems
Source: J Am Chem Soc. 2026 Mar 19;148(12):13160–73. doi: 10.1021/jacs.5c22987 (PMC13047695; doi:10.1021/jacs.5c22987)
Supplement: Supplementary file 1 [file ja5c22987_si_001.pdf]

Supporting information

Integrating NMR restraints into coarse-grained simulations: toward accurate conformational ensembles of complex protein systems

Mina Cullen[1], Carmen Biancaniello[2], Katerina Taškova[3], Vedran Miletić[4], Davide Mercadante[1,5,*], Alfonso De Simone[2,*]

[1]School of Chemical Sciences, The University of Auckland, Auckland, New Zealand.
[2]Dipartment of Pharmacy, University of Naples Federico II, Naples, Italy.
[3]Department of Computer Science, The University of Auckland, Auckland, New Zealand.
[4]Max Planck computing and data facility (MPCDF), Garching, Munich, Germany.
[5]Maurice Wilkins Centre for molecular biodiscovery, The University of Auckland, Auckland, New Zealand.
*Correspondence: alfonso.desimone@unina.it or davide.mercadante@auckland.ac.nz

Table of Contents

1. Supplementary Figures S1-S14………………………………………………………………………………… 2

2. References 17

Supplementary Figures

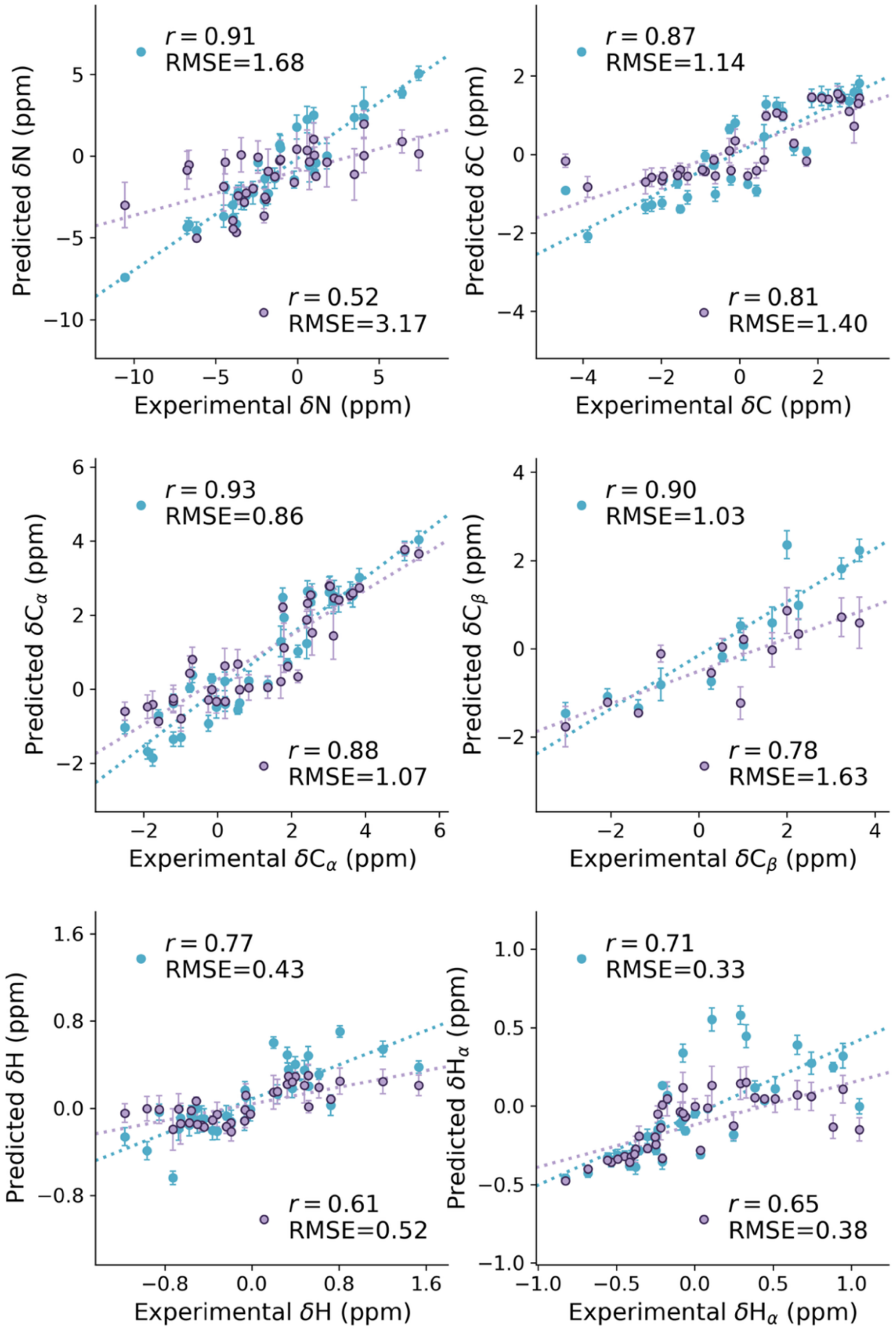

Figure S1 | Agreement between experimental and predicted chemical shifts. Correlation between experimental and computed CS for the backbone atoms from simulations performed on a de novo designed mini-protein (PDB ID: 2ND3). In blue simulations performed using Martini3-NMR and in indigo Martini3-DSSP.

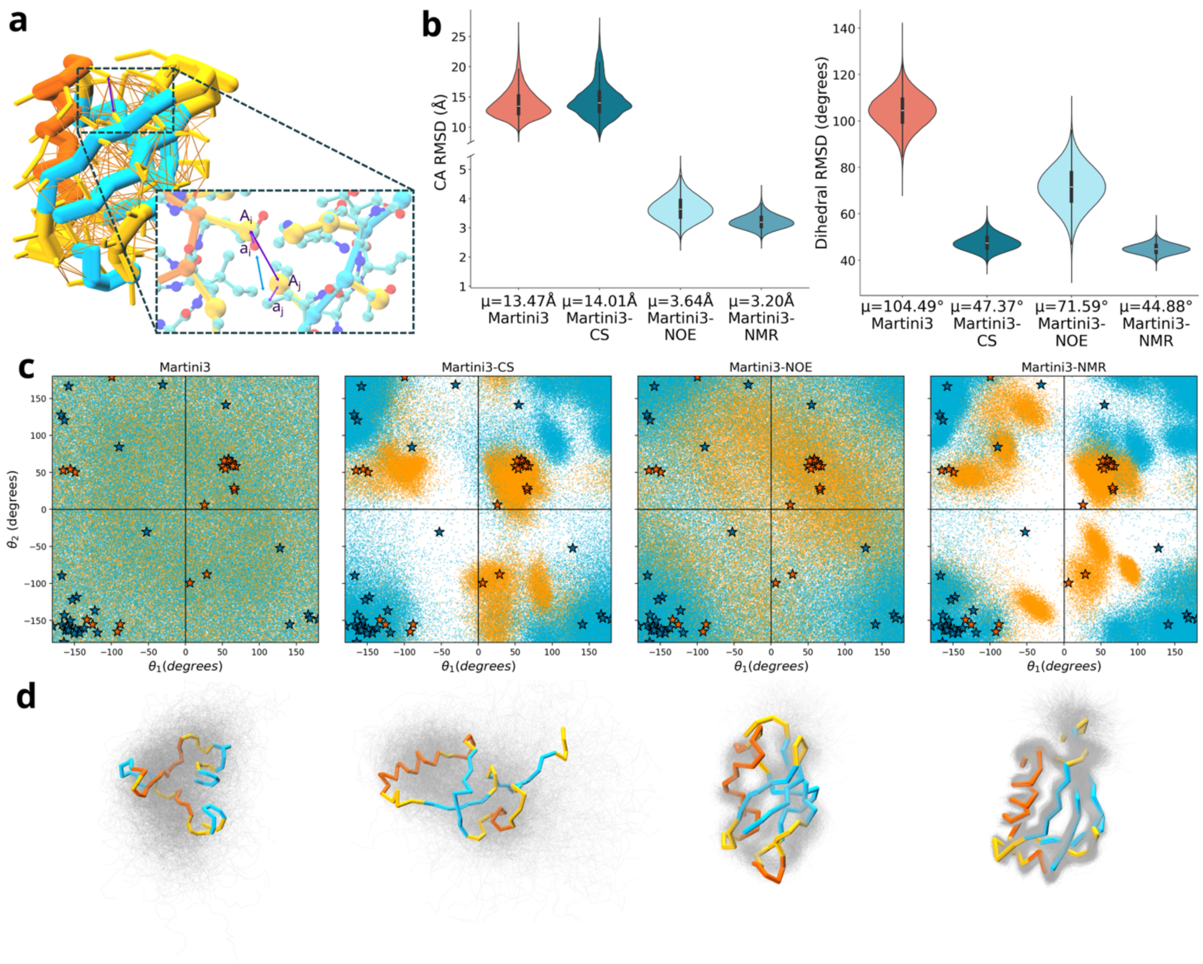

Figure S2. | Effect of CS and NOE restraints in keeping tertiary packing. (a) Schematic representation of NOEs mapping in Martini3-NMR. Orange lines show the mapping of NOEs restraints across ubiquitin (PDB CODE: 1UBQ). The inset shows the CG structure overlaid to an all-atom representation, where NOE restraints are represented by arrows between CG beads mapped on the side chain center of mass. (b) Cα (left) and dihedral (right) root mean square deviation (RMSD) distributions (as violin plots) for simulations of ubiquitin carried out using unrestrained Martini3, only CS restraints (Martini3-CS), only NOE restraints (Martini3-NOE) or CS and NOE restraints (Martini3-NMR). (c) From left to right: pseudo-Ramachandran plots in θ_1 and θ_2 dihedral space describing backbone secondary structure in simulations carried out using unrestrained Martini3, only CS restraints (Martini3-CS), only NOE restraints (Martini3-NOE) or CS and NOE restraints (Martini3-NMR). (d) From left to right, Ubiquitin ensembles obtained from simulations carried out using unrestrained Martini3, only CS restraints (Martini3-CS), only NOE restraints (Martini3-NOE) or CS and NOE restraints (Martini3-NMR).

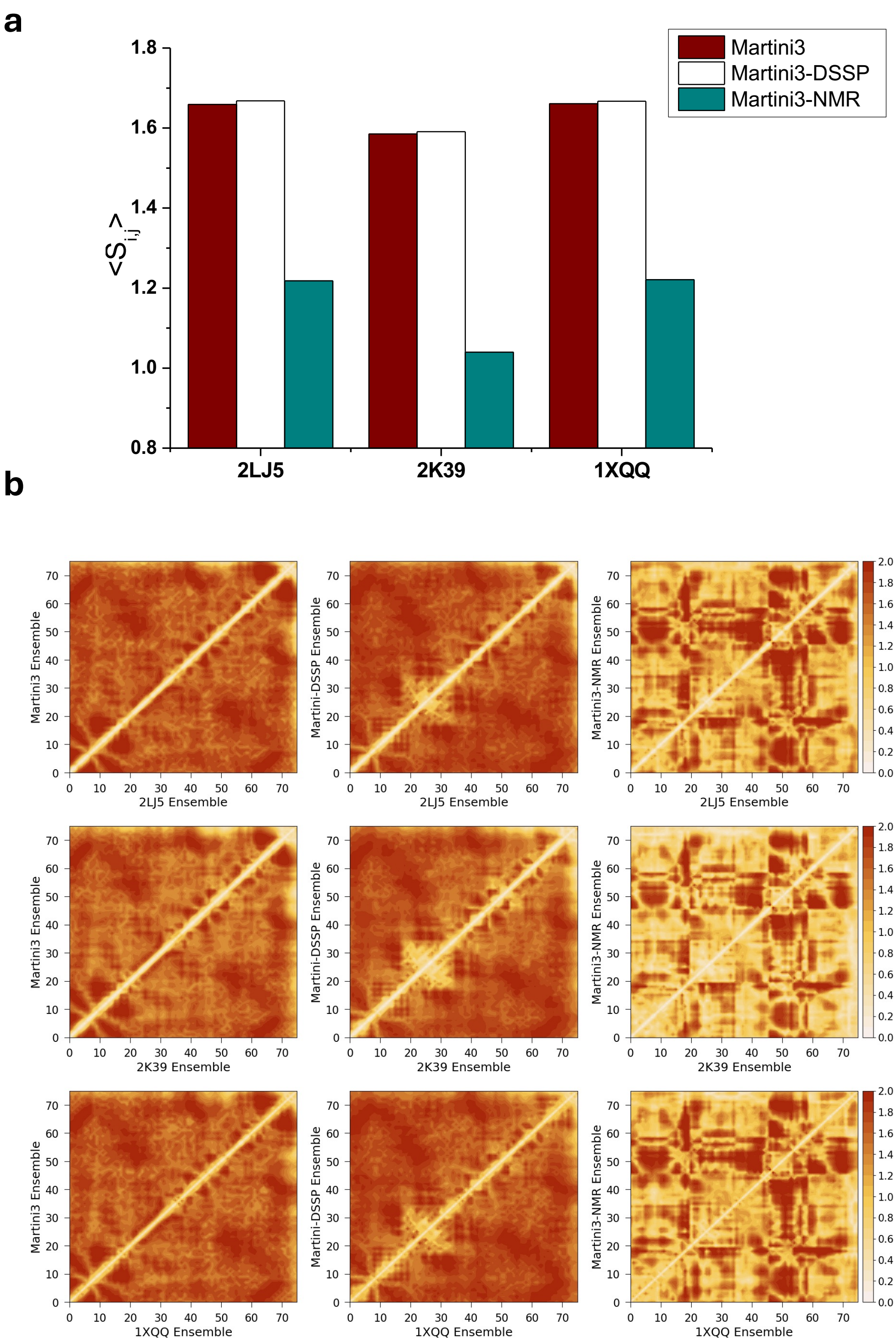

Figure S3 | Analysis of the distributions of interatomic distances in CG ensemble and high-resolution full atomic ensembles of Ubiquitin. The S matrix[1] compares the pairwise distributions in two ensembles thereby accounting for the similarities in both structure and dynamics. In this case the BB-BB distance distributions in the CG ensembles where compared with the Cα-Cα from the full atomic structures. The three CG ensembles where compared with high-resolution structures refined with NMR restraints in full atomic samplings PDB codes 2LJ5[2], 2K39[3] and 1XQQ.[4] Across all systems examined, Martini3-NMR consistently outperformed Martini3 and Martini3-DSSP in reproducing the conformational properties of the full-atom ensembles. **a**) Average $<S_{i,j}>$ values calculated on secondary structure elements with

|i-j|>2. **b)** S matrix plotted as a function of the Ubiquitin sequence and comparing CG ensembles with full atomic structures.

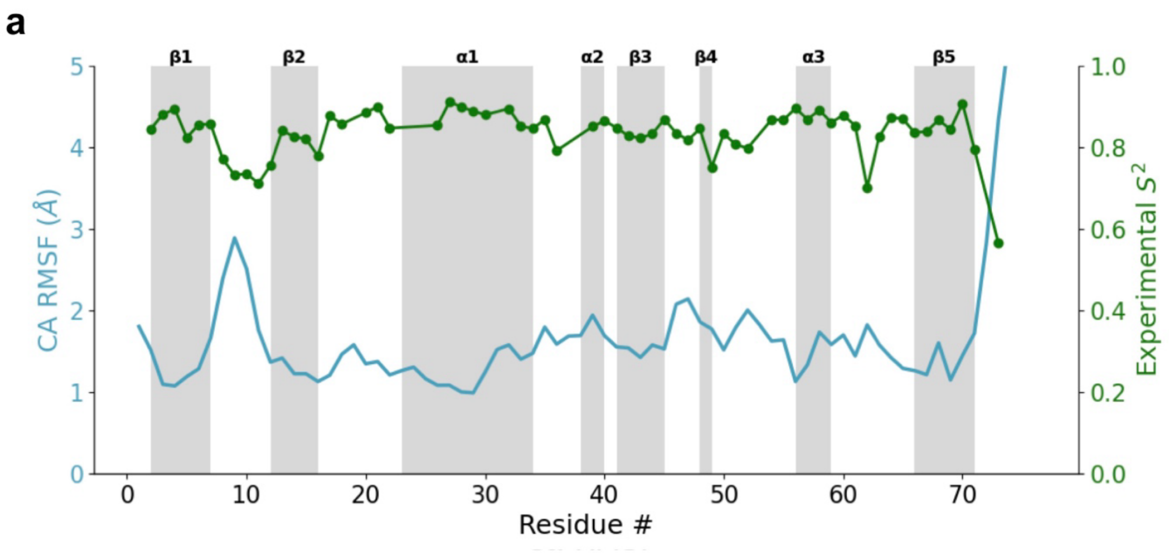

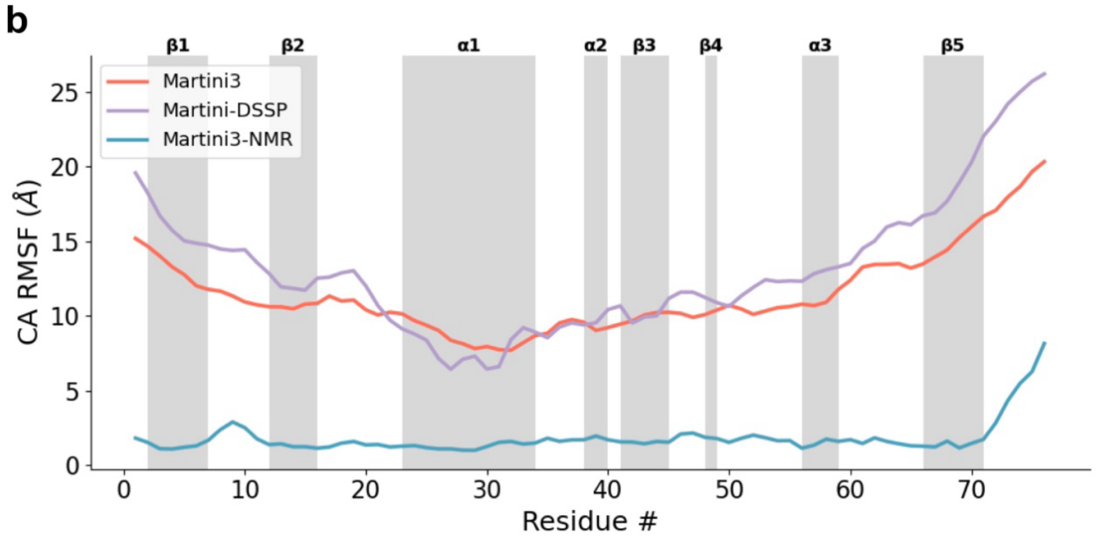

Figure S4 | Root mean square fluctuations (RMSF) for Ubiquitin. RMSF values are calculated for the backbone beads of the Martini3, Martini3-DSSP and Martini3-NMR ensembles of Ubiquitin. **a)** Comparison between RMSF values in the Martini3-NMR ensemble and experimental S^2 from ^{15}N relaxation experiments in solution NMR. The results show excellent correspondence between dynamical regions identified by high RMSF and low S^2 values. **b)** Comparison between RMSF values across the CG ensembles of Ubiquitin.

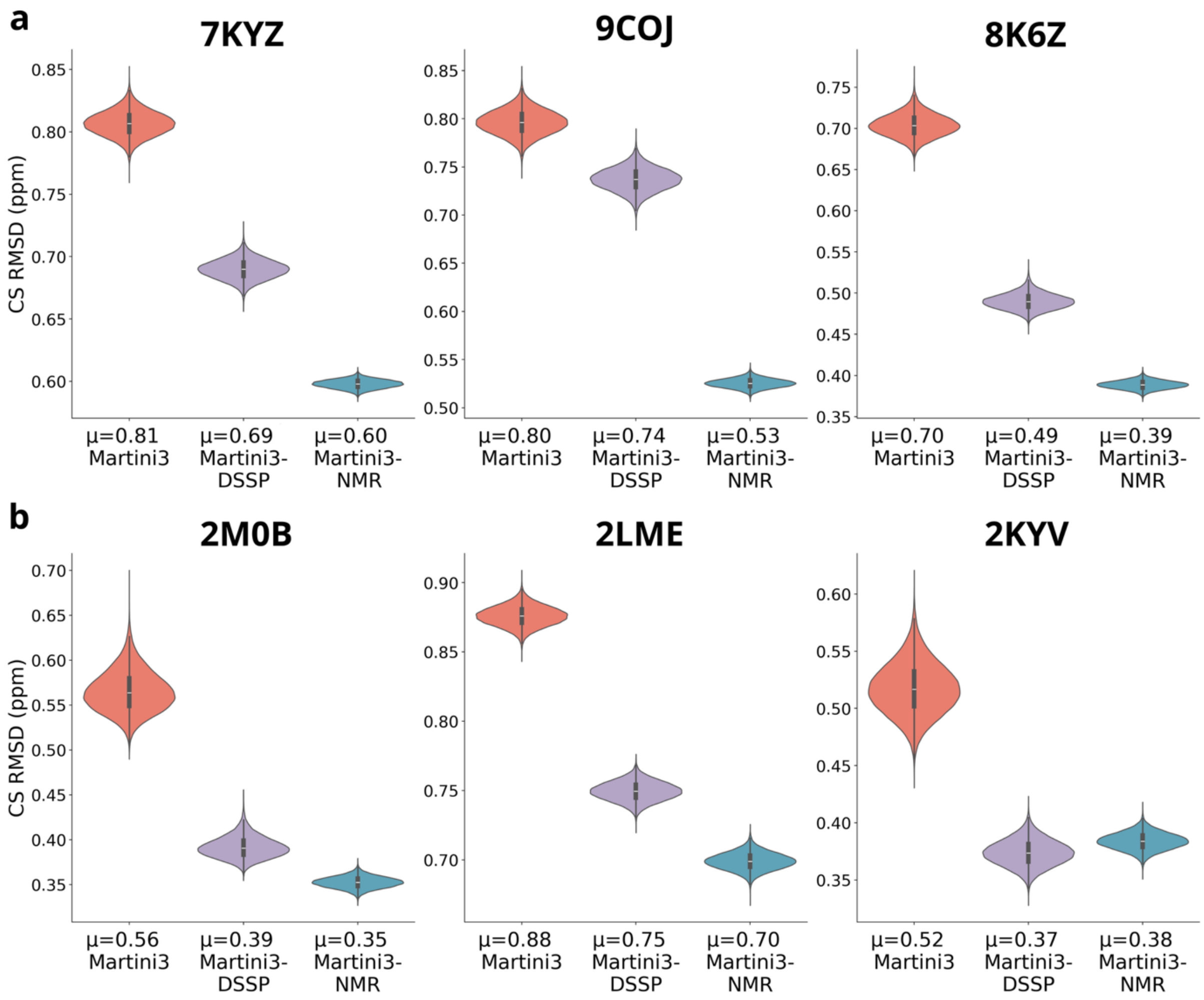

Figure S5. Chemical shift RMSD obtained from simulations of soluble and membrane proteins. (a) Chemical shift RMSD of simulations of soluble proteins. From left to right are KRAS (PDB ID: 7KYZ), the SH3 tandem domains of the human KIN protein (PDB ID: 9COJ) and a human leptin (PDB ID: 8K6Z). (b) Chemical shift RMSD of simulations of membrane proteins. From left to right are the single-span transmembrane helical domains of the human tyrosine kinase ErbB1 (PDB ID: 2M0B), the transmembrane anchor domain of the bacterial autotransporter YadA (PDB ID: 2LME), and the phospholamban pentamer (PDB ID: 2KYV). The distributions reflect the simulations performed with Martini3 (unrestrained, pink salmon), Martini3-DSSP (indigo) and Martini3-NMR (teal).

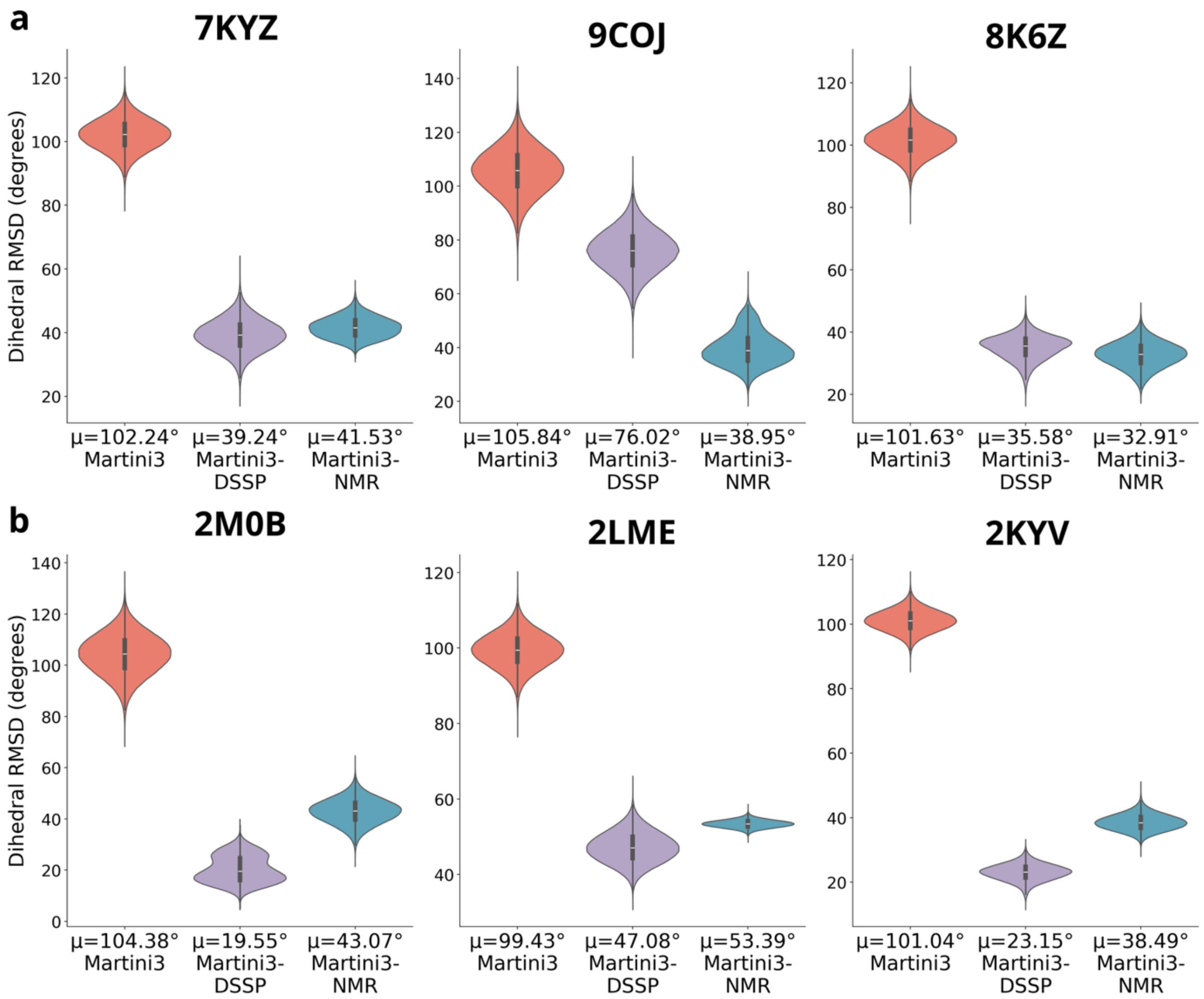

Figure S6 | Dihedral RMSD obtained from simulations of soluble and membrane proteins. (a) Dihedral RMSD of simulations of soluble proteins. From left to right are KRAS (PDB ID: 7KYZ), the SH3 tandem domains of the human KIN protein (PDB ID: 9COJ) and a human leptin (PDB ID: 8K6Z). (b) Dihedral RMSD of simulations of membrane proteins. From left to right are the single-span transmembrane helical domains of the human tyrosine kinase ErbB1 (PDB ID: 2M0B), the transmembrane anchor domain of the bacterial autotransporter YadA (PDB ID: 2LME), and the phospholamban pentamer (PDB ID: 2KYV). The distributions reflect the simulations performed with Martini3 (unrestrained, pink salmon) , Martini3-DSSP (indigo) and Martini3-NMR (teal).

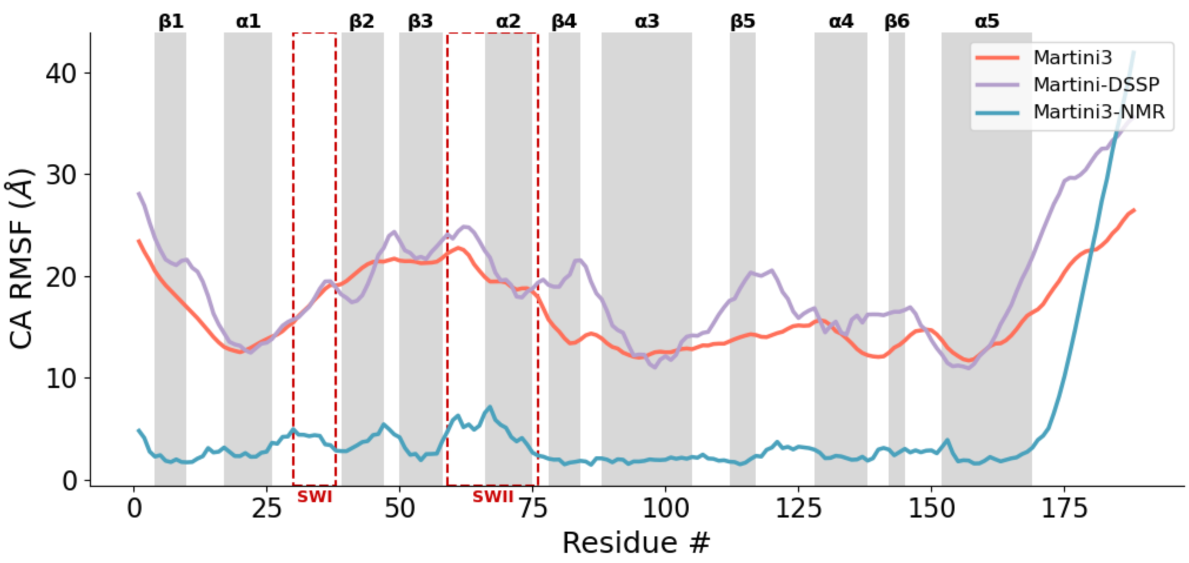

Figure S7 | Root mean square fluctuations (RMSF) for KRAS. RMSF values are calculated for the backbone beads of the Martini3, Martini3-DSSP and Martini3-NMR ensembles of KRAS. While Martini3-NMR yields RMSF values within the range expected for folded proteins, with excellent correspondence to dynamical regions identified by S^2 relaxation measurements (Figure 3b), both Martini3 and Martini3-DSSP exhibit abnormally high RMSF values that fail to reflect the differential flexibility of loops and secondary-structure elements.

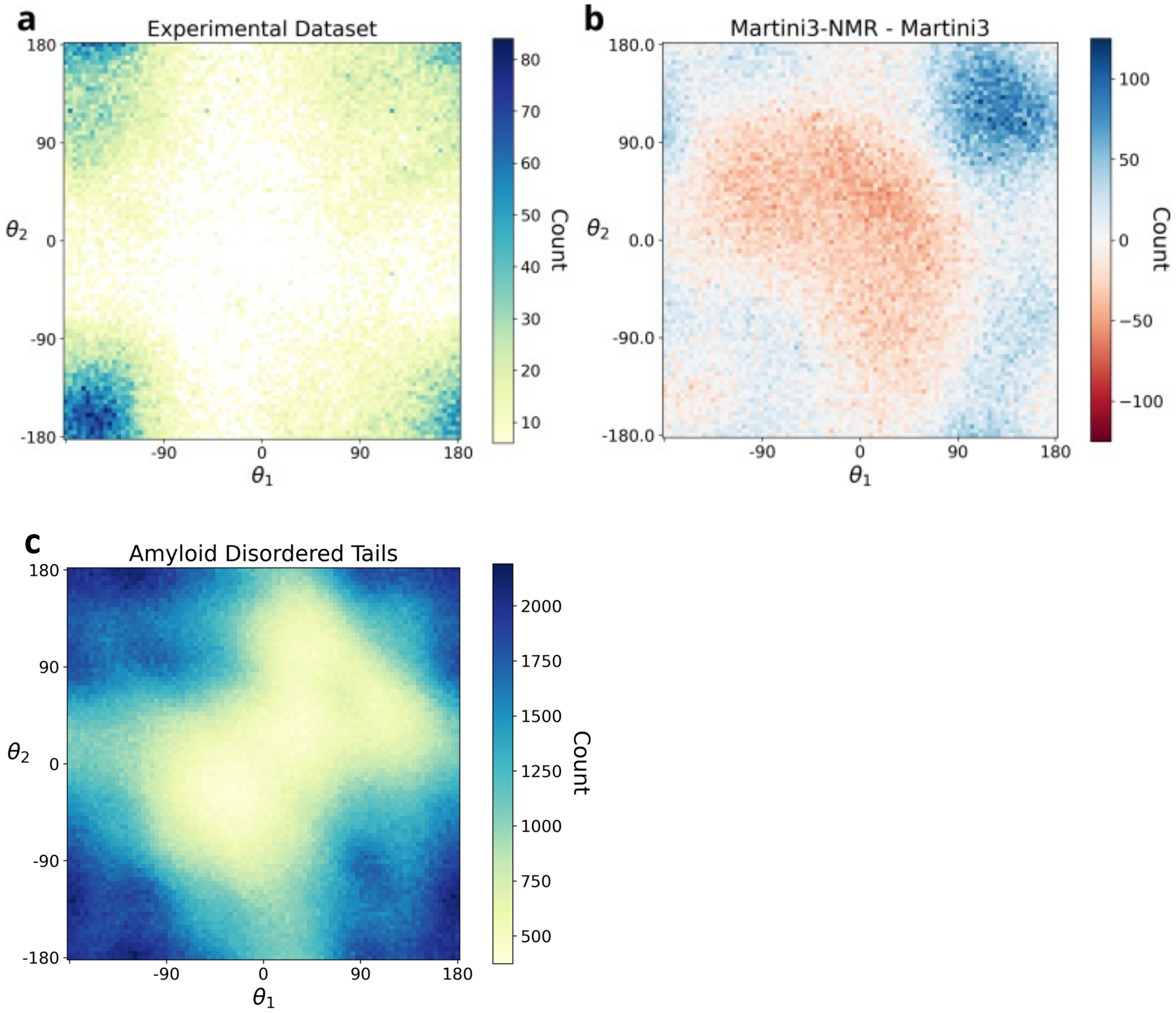

Figure S8 | Dihedral angles in disordered regions of experimental structures and Martini3-NMR ensembles. (a) Pseudoramachandran plot composed of θ_1 and θ_2 angles (Figure 1a) and calculated using the structures of loop regions in folded proteins as employed to train Napshift-CG. (b) Difference between the pseudoramachandran plots computed using Martini3-NMR ensemble of Leptin (disordered loop regions) with respect to Martini3 (unrestrained). Blue points indicate regions of the pseudoramachandran sampled more by Martini3-NMR, while red coloring suggests those regions sampled more by Martini3 unrestrained. The latter fall into forbidden regions of the pseudoramachandran plot as observed in experimental structures, indicating that the NMR restraints have significantly improved the dihedral space with respect to unrestrained simulations. (c) Pseudoramachandran plot of the disordered fuzzy coats in the conformational ensemble of the non-twisting tau-amyloid. The plot shows how the IDRs avoid sampling the forbidden regions of the pseudoramachandran plot centred in θ_1 and θ_2 angle values of 0°.

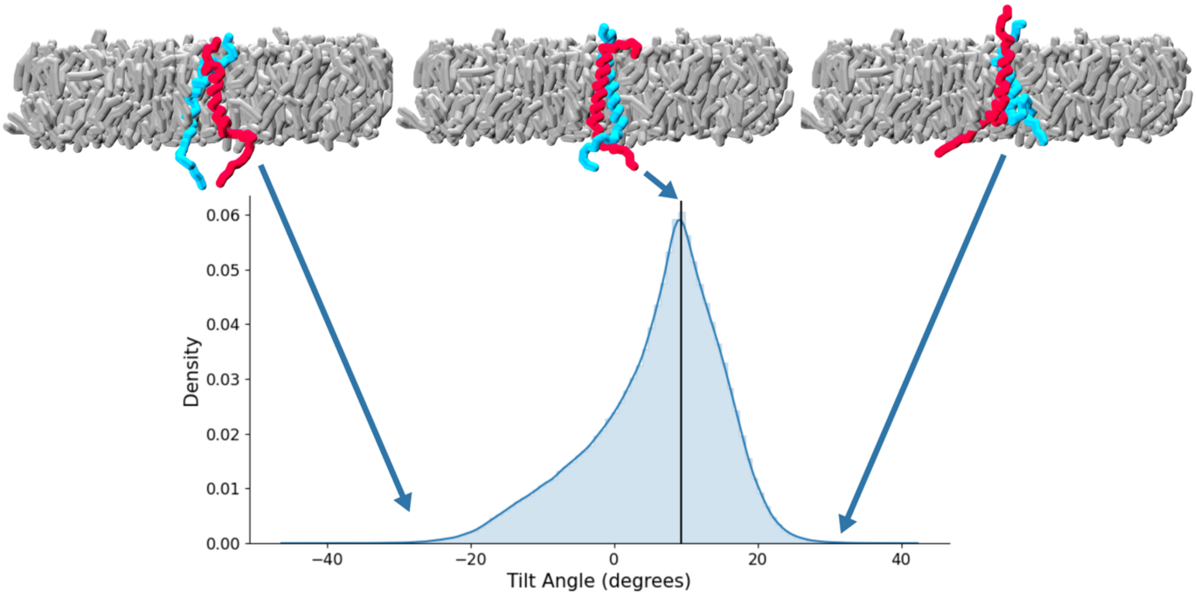

Figure S9 | Orientation of the transmembrane helices of the human tyrosine kinase ErbB1 as sampled by Martini3-NMR. Distribution of the tilt angle describing the relative orientation of the TM helices as sampled by Martini3-NMR. In the top panels ensembles reflective of low, median and high tilt angles as pointed by the arrow. Helices are shown in cyan and red while the membrane is shown in gray.

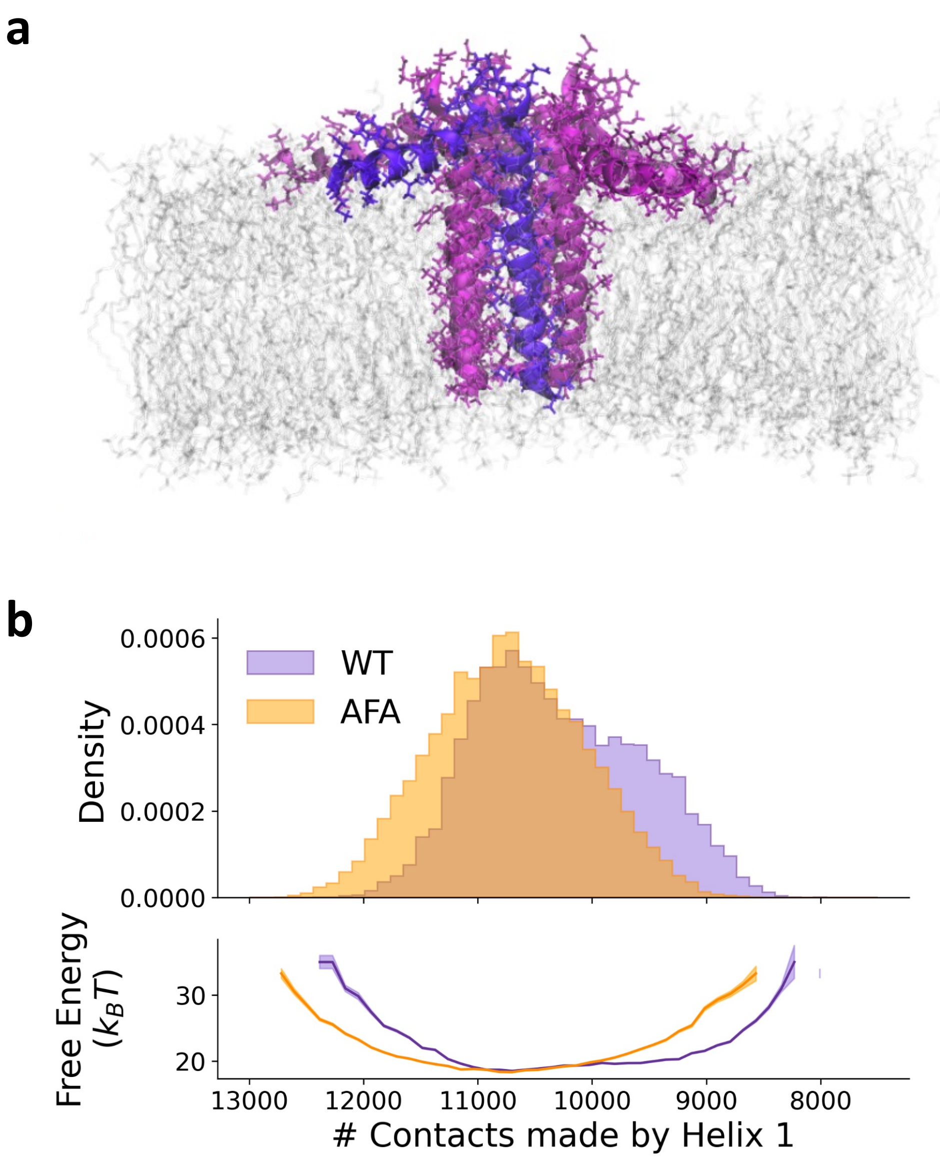

Figure S10 | Phoapholamban pentamer does not dissociate in full-atom restrained simulations. a) To compute the distributions of contacts of helix 1 with the remainder of the pentamer, three full-atom simulations each of 500 ns each, for WT and AFA-mutant phospholamban were performed in explicit membrane and solvent. **b)** Number of contacts between helix 1 and the remainder of the pentamer molecules is shown in indigo and orange for PLN^{WT} and PLN^{AFA}, respectively (top panel). In the bottom panel the free energy landscape along the same reaction coordinate has been computed using the Boltzmann equation. In contrast to Martini3-NMR simulations, the pentamer remains stable in both PLN^{WT} and PLN^{AFA}, emphasising how Martini3-NMR is uniquely able to access to large-scale dissociation processes that are inaccessible in full-atom simulations.

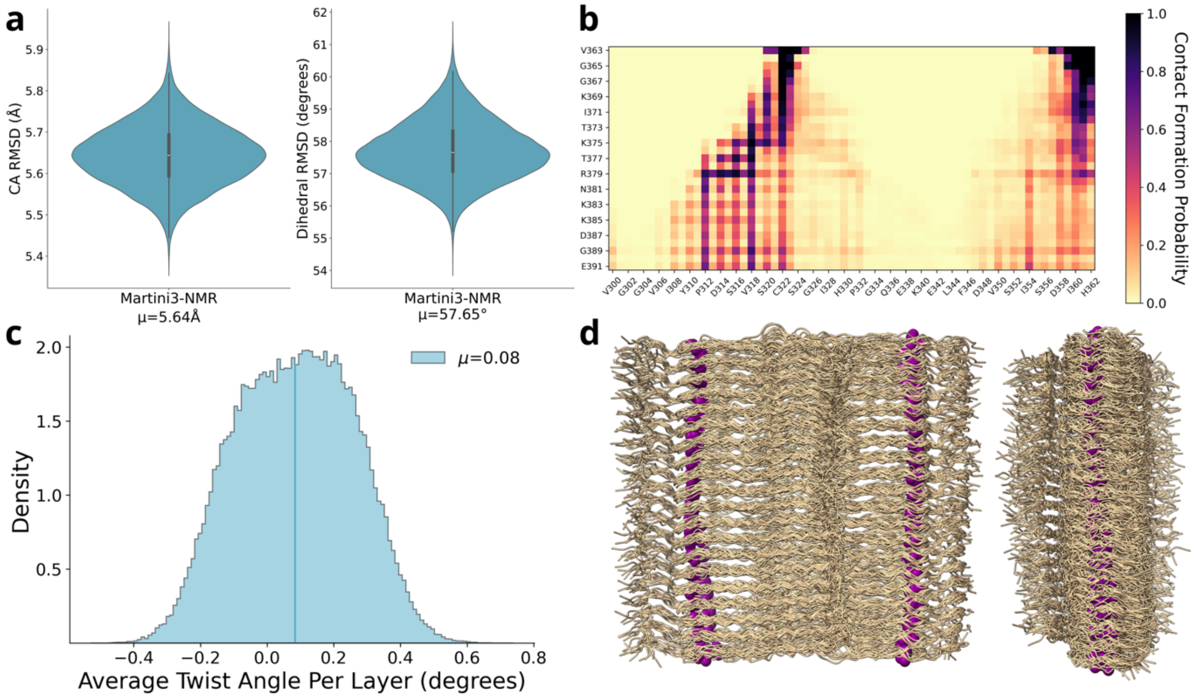

Figure S11 | NMR restrained simulations of the tau amyloid core show minimal twisting when compared to unrestrained simulations. (a) C_α (left) and dihedral (right) RMSD distributions (as violin plots) of the simulations of the tau amyloid (PDB ID: 8G58) carried out either using Martini3-NMR. (b) Fraction of contacts between the intrinsically disordered regions and the core. (c) Distributions of the twisting angle per layer from simulations performed using Martini3-NMR (steel blue). (d) Ensemble of the amyloid core obtained from simulations performed using Martini3-NMR, with the BB beads at the N- and C-terminal residues highlighted as magenta spheres to better track twisting.

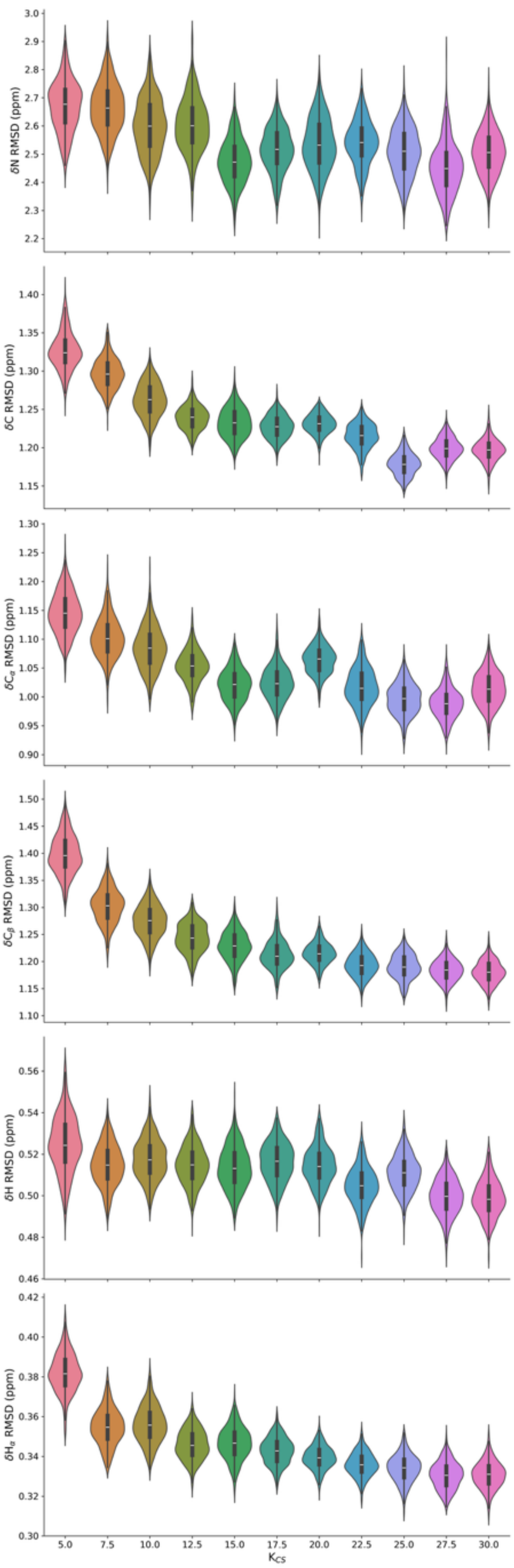

Figure S12 | Scanning of the chemical shift restraints strengths in simulations of ubiquitin. Distributions of chemical shifts RMSD for ubiquitin ensembles obtained at increasing restraints strength, as dictated by the increase in the K parameter (see methods for details). From top to bottom the RMSD is shown for the N, C, C_α, C_β, H and H_α atoms, respectively.

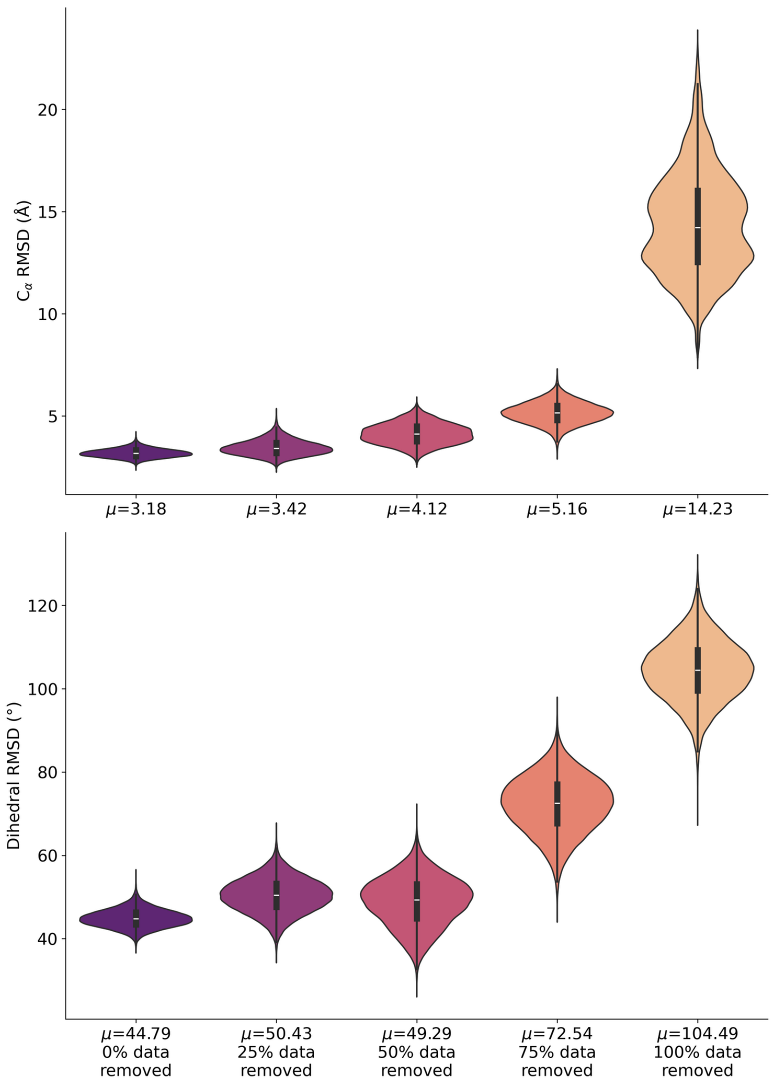

Figure S13

Figure S13 | Ablation study of chemical shift restraints on ubiquitin. (a) Cα and dihedral RMSD distributions of molecular simulations of ubiquitin with several degree of restraints applied. The distributions show the RMSD obtained for ensembles with increasing amount of chemical shifts restraints randomly removed from the experimental set.

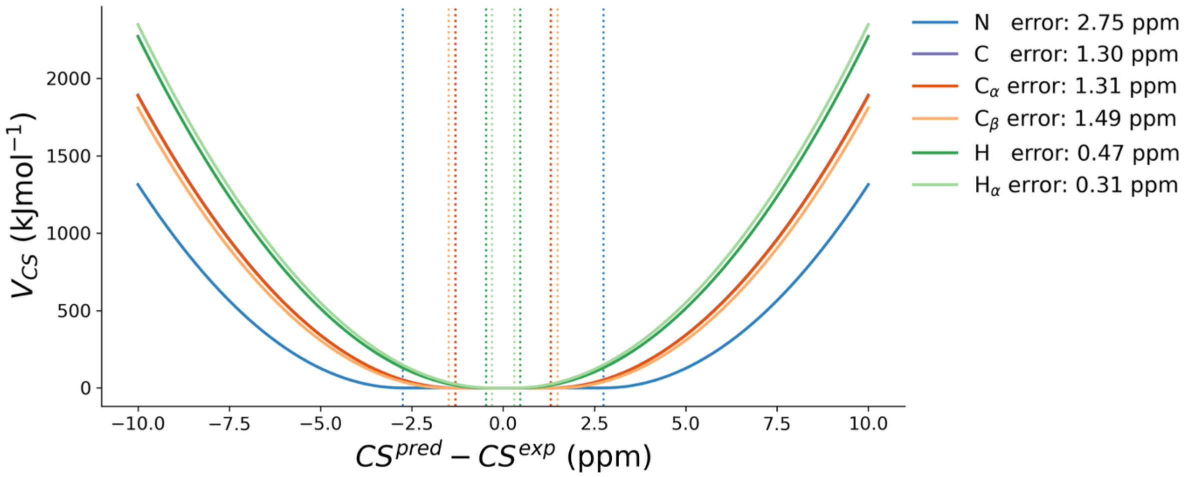

Figure S14 | Flat-bottom chemical shift restraint potential for different atom types. Plots demonstrating [VCS] as a function of the difference between simulated and experimental chemical shifts with [KCS=25] for the 6 atom types. Dashed lines indicate the boundaries of the flat-bottom regime for each atom type.

Supplementary references

(1) De Simone, A.; Richter, B.; Salvatella, X.; Vendruscolo, M. Toward an Accurate Determination of Free Energy Landscapes in Solution States of Proteins. *J. Am. Chem. Soc.* 2009, *131* (11), 3810–3811. https://doi.org/10.1021/JA8087295/SUPPL_FILE/JA8087295_SI_001.PDF.

(2) Montalvao, R. W.; De Simone, A.; Vendruscolo, M. Determination of Structural Fluctuations of Proteins from Structure-Based Calculations of Residual Dipolar Couplings. *J. Biomol. NMR* 2012, *53* (4), 281–292. https://doi.org/10.1007/S10858-012-9644-3/FIGURES/6.

(3) Lange, O. F.; Lakomek, N. A.; Farès, C.; Schröder, G. F.; Walter, K. F. A.; Becker, S.; Meiler, J.; Grubmüller, H.; Griesinger, C.; De Groot, B. L. Recognition Dynamics up to Microseconds Revealed from an RDC-Derived Ubiquitin Ensemble in Solution. *Science (1979).* 2008, *320* (5882), 1471–1475. https://doi.org/10.1126/SCIENCE.1157092/SUPPL_FILE/LANGE.SOM.PDF.

(4) Lindorff-Larsen, K.; Best, R. B.; DePristo, M. A.; Dobson, C. M.; Vendruscolo, M. Simultaneous Determination of Protein Structure and Dynamics. *Nature 2004 433:7022* 2005, *433* (7022), 128–132. https://doi.org/10.1038/nature03199.
